# Therapeutic administration of a recombinant human monoclonal antibody reduces the severity of chikungunya virus disease in rhesus macaques

**DOI:** 10.1371/journal.pntd.0005637

**Published:** 2017-06-19

**Authors:** Rebecca Broeckel, Julie M. Fox, Nicole Haese, Craig N. Kreklywich, Soila Sukulpovi-Petty, Alfred Legasse, Patricia P. Smith, Michael Denton, Carsten Corvey, Shiv Krishnan, Lois M. A. Colgin, Rebecca M. Ducore, Anne D. Lewis, Michael K. Axthelm, Marie Mandron, Pierre Cortez, Jonathan Rothblatt, Ercole Rao, Ingo Focken, Kara Carter, Gopal Sapparapau, James E. Crowe, Michael S. Diamond, Daniel N. Streblow

**Affiliations:** 1 Vaccine and Gene Therapy Institute, Oregon Health and Science University, Beaverton, United States of America; 2 Departments of Medicine, Molecular Microbiology, Pathology & Immunology, Washington University School of Medicine, St. Louis, United States of America; 3 Division of Pathobiology and Immunology, Oregon National Primate Research Center, Beaverton, United States of America; 4 Sanofi, Cambridge, United States of America; 5 Pathology Services Unit, Division of Comparative Medicine, Oregon National Primate Research Center, Beaverton, United States of America; 6 Sanofi, Marcy L’Etoile, France; 7 Departments of Pediatrics and Pathology, Microbiology, and Immunology, Vanderbilt University, Nashville, United States of America; Centers for Disease Control and Prevention, UNITED STATES

## Abstract

Chikungunya virus (CHIKV) is a mosquito-borne virus that causes a febrile syndrome in humans associated with acute and chronic debilitating joint and muscle pain. Currently no licensed vaccines or therapeutics are available to prevent or treat CHIKV infections. We recently isolated a panel of potently neutralizing human monoclonal antibodies (mAbs), one (4N12) of which exhibited prophylactic and post-exposure therapeutic activity against CHIKV in immunocompromised mice. Here, we describe the development of an engineered CHIKV mAb, designated SVIR001, that has similar antigen binding and neutralization profiles to its parent, 4N12. Because therapeutic administration of SVIR001 in immunocompetent mice significantly reduced viral load in joint tissues, we evaluated its efficacy in a rhesus macaque model of CHIKV infection. Rhesus macaques that were treated after infection with SVIR001 showed rapid elimination of viremia and less severe joint infiltration and disease compared to animals treated with SVIR002, an isotype control mAb. SVIR001 reduced viral burden at the site of infection and at distant sites and also diminished the numbers of activated innate immune cells and levels of pro-inflammatory cytokines and chemokines. SVIR001 therapy; however, did not substantively reduce the induction of CHIKV-specific B or T cell responses. Collectively, these results show promising therapeutic activity of a human anti-CHIKV mAb in rhesus macaques and provide proof-of-principle for its possible use in humans to treat active CHIKV infections.

## Author summary

Chikungunya virus (CHIKV) causes fever, rash, and acute and chronic arthralgia. Currently there are no approved therapies to treat or vaccines to prevent CHIKV infection in humans. In this study, we engineered SVIR001, a recombinant fully human monoclonal antibody (mAb) that eliminated viremia, reduced viral load at the site of infection, and diminished spread to distant target tissues in rhesus macaques when administered after infection. SVIR001 treatment reduced joint inflammation and disease without impairing the induction of the adaptive immune response. These results demonstrate the efficacy of mAb therapy to reduce the severity of CHIKV disease.

## Introduction

Chikungunya virus (CHIKV) is a mosquito-transmitted, positive-sense, enveloped RNA virus. The mature virion has an icosahedral surface studded with viral glycoproteins E1 and E2 and a central capsid core, which encapsulates viral RNA [1]. CHIKV causes severe, debilitating joint and musculoskeletal disease that may persist for months to years following acute infection [2–4]. Over the past decade, CHIKV has caused several large-scale epidemics and expanded its geographic range to five continents. During the 2004–2007 Indian Ocean Island outbreak, CHIKV emerged from Kenya to several islands in the Indian Ocean, where infection attack rates reached as high as 75% of the population [5–7], and to India, where a suspected 1.3 million people were infected during 2006 [8]. From India, the virus spread to China and Southeast Asia [9]. At the end of 2013, local transmission of CHIKV occurred in the Caribbean island of Saint Martin [10]. CHIKV disseminated rapidly throughout the Caribbean and to neighboring countries in Central and South America, infecting an estimated 1.8 million people in more than 40 countries [11]. The recent outbreak of CHIKV in the Americas raises concern about its continued spread in this region and its introduction into new areas with large naïve populations.

In humans, CHIKV infection symptoms appear within 3 to 12 days after the bite of an infected mosquito [12]. During the acute phase, humans experience arthralgias, high-grade fever, skin rash, and headaches. CHIKV-induced polyarthralgia can be debilitating, affecting many peripheral joints including the ankles, knees, wrists, and fingers [13]. The elderly (>60 years) and newborns typically sustain higher viral loads [14] and are at increased risk for severe disease manifestations such as encephalitis, seizures, cardiovascular complications, and death [15, 16]. During the convalescent phase of the disease, viremia, fever and rash resolve although joint and muscle pain may linger for months to years [3, 17]. Continued long-term arthralgias is a common symptom, with one population-based survey reporting that 43–75% of patients experienced long-term joint pain for at least two years following CHIKV infection [18]. Although infectious virus has not been recovered from joints of patients experiencing chronic pain, CHIKV RNA and antigen were detected in muscle and synovial biopsies months after the resolution of the acute symptoms [3, 19]. These results suggest that CHIKV may establish a persistent infection in musculoskeletal tissue, which results in chronic joint and muscle pain. Currently there are no approved vaccines or therapies for the prevention or treatment of CHIKV.

Many of the candidate CHIKV vaccines under development rely on the induction of neutralizing antibodies for protection against challenge [20–22]. In humans, prior infection and development of anti-CHIKV neutralizing antibodies have been associated with protection from reinfection, further supporting the development of antibody-based vaccines and therapeutics [23–25]. CHIKV-specific IgM can be detected by day 4 of illness and CHIKV-specific IgG antibodies can be detected by day 10 [25, 26]. The antibody response following CHIKV infection is directed primarily against viral glycoprotein E2 and is long-lived [27]. Anti-CHIKV antibodies generated following CHIKV infection possess broad neutralizing activity against viruses belonging to different CHIKV genotypes [27, 28], and antibodies generated following infection with closely related non-CHIKV Alphaviruses could have cross-neutralizing activity against CHIKV [29]. Since CHIKV naturally infects nonhuman primates (NHPs) during sylvatic transmission cycles, NHPs are a relevant animal model for testing CHIKV vaccines and therapeutics and studying immunity. NHPs also develop antibodies directed against CHIKV following infection; mapping of the linear B cell epitopes showed that these antibodies were directed against several CHIKV proteins, including E2 [26, 30].

Antibody-mediated protection against CHIKV infection has been reported in animal models. Passive transfer of immune sera into susceptible neonatal mice as well as *Ifnar*-/- and AG129 mice protects against lethal CHIKV challenge [31–34]. Mice deficient in B cells (μMT) develop a nonlethal infection with persistent viremia [35, 36], which can be prevented by passive transfer of serum-derived anti-CHIKV polyclonal antibodies [37]. Neutralizing mouse monoclonal antibodies (mAbs) have protective efficacy in animal models when administered as post-exposure therapy [31, 32]. Our group previously established the utility of mAb therapy for CHIKV in *Ifnar*-/- mice and in rhesus macaques (*Macaca mulatta*) [32, 38]. In *Ifnar*-/- mice, passive transfer of a combination of two mouse mAbs (CHK-152 and CHK-166) against E2 and E1 proteins increased survival when given up to 60 hours post-infection. In rhesus macaques, CHK-152 and CHK-166 combination therapy at days 1 and 3 of CHIKV infection resulted in elimination of viremia and reduction of viral spread to joints and muscles of the legs. However, the viral load at the site of infection and in the draining lymph nodes were unchanged relative to controls at 7 days post infection (dpi). These results suggested that a CHIKV mAb therapeutic required further optimization to control acute infection, minimize viral persistence, and prevent long-term joint disease.

To this end, we recently isolated 4N12, a highly neutralizing human mAb, from a subject who had a history of CHIKV infection 5.5 years prior to blood donation [28]. This mAb exhibited high levels of protection in *Ifnar-/-* mice, even when administered as monotherapy up to 60 hours after CHIKV infection [28]. In this current study, we engineered the mAb SVIR001, which was derived from the parent mAb 4N12, and characterized its therapeutic potential. SVIR001 was as effective in neutralizing CHIKV as the parent 4N12, and therapeutic administration of SVIR001 reduced viral burden in joint tissues of wild-type (WT) immunocompetent mice. In rhesus macaques, SVIR001 treatment at days 1 and 3 after infection eliminated viremia and infectious virus from tissues. SVIR001 reduced CHIKV infection at the inoculating site and blocked spread to and inflammation within distant tissue sites including joints and muscles. These results in rhesus macaques suggest the potential for SVIR001 mAb therapy to reduce CHIKV-associated inflammatory disease in humans.

## Methods

### Ethics statement

All experiments involving rhesus macaques and mice were performed in compliance with good animal practices as outlined in local and national animal welfare bodies. Rhesus macaque studies were performed in the ABSL-3 containment facility at the Oregon National Primate Research Center (ONPRC). Mouse experiments were performed at Washington University School of Medicine in an approved ABSL-3 facility (IACUC #20140199). Both facilities are accredited by the Assessment and Accreditation of Laboratory Animal Care (AAALAC) International. All experiments were performed in strict accordance to Institutional Animal Care and Use Committee (IACUC) protocols (IACUC #0993). Appropriate procedures were utilized in order to reduce potential distress, pain and discomfort. For example, ketamine (10 mg/kg) was used to sedate the Rhesus macaques during all procedures including routine blood draws performed by trained veterinary staff. The animals were fed standard monkey chow with routine food supplements for enrichment. The infected animals were caged with partners or separately but within visual and auditory contact of other animals in order to promote social behavior. Animals were euthanized according to the recommendations of the American Veterinary Medical Association 2013 Panel on Euthanasia.

### Cells and viruses

The infectious clone of CHIKV-LR (CHIKV LR2006 OPY1) was provided generously by Steven Higgs (Kansas State University, Manhattan, KS). Viral stocks were propagated in BHK21 cells and passaged in C6/36 *Aedes albopictus* cells. The infectious clone of CHIKV-181/25 was provided generously by Terence Dermody (University of Pittsburgh, Pittsburgh, PA). Stocks were propagated in BHK21 cells or Vero cells. CHIKV plaque or focus-forming assays were performed on Vero cell culture monolayers, as previously described [32, 38]. All cells were cultured in Dulbecco’s Modified Eagle Medium (DMEM) supplemented with 5–10% fetal bovine serum (FBS) and penicillin-streptomycin-glutamine. Vero and BHK21 cell monolayer cultures were grown at 37°C with 5% CO_2_, and C6/36 cells were grown at 28°C with 5% CO_2_.

### Neutralization assays

Neutralization assays with CHIKV were performed as previously described [32]. Approximately 100 focus-forming units (FFU) of CHIKV-LR were incubated with serial dilutions of mAbs for 1 h at 37°C and then plated onto Vero cells. Plates were incubated for 90 minutes at 37°C and overlaid with a carboxy methylcellulose-containing medium. At 18 h post-infection, plates were fixed with 1% paraformaldehyde in PBS, and infectious virus was measured by a FFU assay. Plates were incubated with a detection anti-CHIKV mouse mAb (CHK-11 [32]) diluted in permeabilization solution containing 0.1% saponin and 0.1% bovine serum albumin followed by an anti-mouse horseradish peroxidase-conjugated secondary antibody. Foci were visualized using the TrueBlue peroxidase substrate (KPL) and quantified with an ImmunoSpot 5.0.37 microanalyzer (Cellular Technologies Ltd).

### Human mAbs

SVIR001 is a recombinant, fully human IgG1 antibody that recognizes the E2 protein of CHIKV. The complementarity determinant region sequence of the antibody was derived from the human hybridoma mAb 4N12 [28]. SVIR002 is a recombinant isotype control human IgG1 mAb directed against chicken lysozyme. Recombinant mammalian cell-expressed SVIR001 and SVIR002 antibodies were produced at Evitria AG (Wagistrasse 27, 8952 Zurich-Schlieren, Switzerland). The antibody heavy-chain and light-chain cDNAs were cloned into Evitria's evi-5 mammalian gene expression vector. The antibodies were produced by CHO-evi cells, which are Chinese hamster ovary (CHO) K1 cells adapted to serum-free growth in suspension culture. The seed culture was grown in eviGrow medium, a chemically defined, animal component-free, serum-free cell culture medium. Transfection and antibody expression were performed in eviMake at 37°C and 5% CO_2_ using eviFect as the transfection reagent. The cell culture supernatant was harvested by centrifugation eight days after transfection and sterile filtered (0.2 μm). The antibodies were purified by MabSelect SuRe affinity chromatography using Dulbecco's PBS as the wash buffer and 0.1 M glycine, pH 3.0–3.5 as the elution buffer. The purified antibodies were dialyzed against PBS, sterile filtered, and stored at 4°C. Antibody purity was assessed by polyacrylamide gel electrophoresis (Bio-Rad Experion system) under non-reducing and reducing conditions and determined to be greater than 95% for both SVIR001 and SVIR002. The percentage of the total antibody present in an aggregated form, determined by analytical gel filtration chromatography (SEC-HPLC), was less than 0.8% for SVIR001 and less than 0.1% for SVIR002. Endotoxin was determined with the Charles River Endosafe-PTS system and found to be less than 1 EU per milligram of antibody.

### Generation of SVIR001 escape mutant CHIKV

A CHIKV-181/25 strain SVIR001 escape variant virus was generated by repeated passaging in the presence of mAb, as previously described [32]. For the first passage, 1.2 x 10^5^ FFU of CHIKV-181/25 was incubated with 10 μg/ml of SVIR001 for 1 h at 37°C prior to inoculation in Vero cells. After 24 hrs, half of the supernatant was removed and incubated with 10 μg/ml SVIR001 for 1 h, and these complexes were used to inoculate fresh Vero cell monolayer cultures. Following six passages with SVIR001, viral RNA was harvested using QIAamp viral RNA mini kit (Qiagen) and cDNA was generated with random hexamers and Superscript III RT (Invitrogen). The cDNA was amplified by PCR and the E2 and E1 genes were sequenced to identify the genetic change associated with the mAb resistant phenotype. A list of primers used for sequencing are provided in **S1 Table**. A deletion of E2 nucleotides 734–739 was identified and the deletion was engineered into the CHIKV-181/25 and CHIKV-LR infectious clones using Phusion high fidelity DNA polymerase (New England Biolabs). A list of mutagenesis primers are provided in **S2 Table**. The cycling times were 98°C for 30 sec, 18 cycles of 98°C for 30 sec, 50°C for 30 sec, 72°C 7 min with a final extension at 72°C for 10 min. The parental plasmid was digested with DpnI at 37°C for 3 h and the mutant plasmid was transformed into XL-Gold Ultracompetent cells (Agilent). Bacteria were plated onto LB agar supplemented with 100 μg/ml of carbenicillin. Deletion was confirmed by sequencing of the plasmid. WT and mutant CHIKV were produced after plasmid linearization with NotI (New England Biolabs) and *in vitro* transcription with SP6 RNA polymerase (Ambion) following the manufacturer’s instructions. RNA was electroporated into BHK21 cells using a 2 mm cuvette with 2 pulses (850 V, 25 μF, and infinite resistance). Virus was collected from the supernatant of transfected cells 40 h later. To confirm stability of the mutation, RNA was isolated from virus stocks. cDNA was produced using Superscript III First Strand Synthesis system (Invitrogen) and amplified using primers listed in **S1 Table** or previously described [32] and sequenced directly.

### Mouse experiments

CHIKV infections were performed in 4 week-old C57BL/6J WT mice. For the infection studies, WT mice were inoculated with 10^3^ FFU of CHIKV-LR subcutaneous (s.c.) in the footpad. At times indicated, the mice were administered 50 or 300 μg anti-CHIKV mAb SVIR001 or isotype control antibody SVIR002 via an intraperitoneal route. At 3, 5 or 28 dpi, animals were sacrificed and ankle tissues were homogenized. Ankle viral load was measured by focus forming unit assays on Vero cells or by a TaqMan-based quantitative real time reverse transcription PCR assay (qRT-PCR, see below).

### NHP experiments

Each of twelve male rhesus macaques was inoculated subcutaneously with 10^7^ PFU of CHIKV-LR diluted in 1 ml of PBS that was distributed over both hands and arms as ten 100 μl injections as previously described [38]. At 1 and 3 dpi monkeys were infused intravenously with either control mAb (SVIR002) at 15 mg/kg or anti-CHIKV mAb (SVIR001) at 5 or 15 mg/kg (see **Table 1** for specific animal groupings). The mAbs were diluted with saline into a total of 20 mls and infused at 1 ml/minute using a medical syringe pump delivery system. Peripheral blood samples were obtained at 0, 1, 2, 3, 4, 5 and 7 dpi. Whole blood was centrifuged over lymphocyte separation medium (Corning) for 45 min at 3,000 rpm (1,459 x *g*) to isolate peripheral blood mononuclear cells (PBMCs) and plasma. PBMCs were analyzed for lymphocyte phenotype and frequency by flow cytometry, and plasma was assessed for viral load by qRT-PCR and levels of cytokines by a Monkey Magnetic 29-plex Panel for Luminex Platform Kit (Invitrogen). Animals were euthanized at 7 dpi and complete necropsies were performed. Representative tissue samples from (joints, muscles, organs, brain, lymph nodes and bone marrow) were collected and stored in RNA*later*, Trizol (RNA isolation), medium (virus isolation), and 10% buffered formalin (microscopic evaluation).

**Table 1:** CHIKV isolation from tissue homogenates.

mAb	Dose (mg/kg)	Animal	Tissue Virus Isolation
**SVIR002** **(anti-lysozyme control)**	15	31559	Spleen, Finger Joints
		31055	Finger Joints
		31289	Wrist Joints
		31333	Elbow & Wrist Joints, Hamstring
**SVIR001** **(anti-CHIKV)**	15	31078	Negative
		31296	Negative
		31312	Negative
		31302	Negative
	5	31088	Negative
		31335	Negative
		30430	Negative
		31651	Negative

Three groups of four animals each were treated with control antibody SVIR002 at 15 mg/kg, SVIR001 at 15 mg/kg, or SVIR001 at 5 mg/kg. To isolate CHIKV from the infected joint and muscle tissues an aliquot of tissue homogenate was incubated in C6/36 cells for four days. Following incubation, infectious virus was recovered by plaque assay on Vero cells. Only the virus isolation-positive tissues are listed. The limit of detection was 1 PFU per ml of C6/36 supernatant.

### Pharmacokinetic analysis of mAb

Plasma mAb levels were measured by enzyme-linked immunosorbent assay (ELISA). High-binding polystyrene ELISA plates (Corning) were coated with 100 μl of 10^7^ PFU/ml of CHIKV 181/25 to measure SVIR001 levels or 1 μg/ml of lysozyme (Fisher) in PBS to measure SVIR002 levels. Plates were blocked with 2% milk in 0.05% Tween-PBS followed by incubation with plasma dilutions in milk/Tween buffer for 2 h. Plates were washed with 0.05% Tween-PBS and incubated with peroxidase-conjugated anti-human IgG gamma chain (Rockland). Bound antibody was detected using the o-phenylenediamine dihydrochloride (OPD) substrate (Life Technologies), and the plates were read with a Synergy HTX Microplate Reader (BioTek) at 490 nm. A standard curve was generated using serial dilutions of SVIR001 or SVIR002. Plasma antibody concentrations were calculated using the standard curve and multiplied by the dilution factor.

### qRT-PCR analysis

CHIKV RNA levels were measured using a published qRT-PCR assay [38, 39]. Rhesus macaque tissues were homogenized in 1 ml of TRIzol reagent plus approximately 250 μl of SiLiBeads, type S (1.7 to 2.1 mm), using a Precellys 24 homogenizer (Bertin Technologies). Total RNA from tissue samples was prepared using Trizol. Total RNA was prepared from 200 μl of plasma using the Viral RNA kit (Zymol). The isolated RNA was quantified using a Nanodrop spectrophotometer. RNA was treated with RNase-free DNase, and then single-stranded cDNA was generated from 1 μg of RNA using random hexamers and Superscript III RT (Invitrogen). Gene amplicons or viral RNA isolated from infectious virus served as quantification standards (sensitivity, 10 to 100 copies). Quantitative RT-PCR was performed and analyzed using ABI StepOne Plus real-time PCR system (Applied Biosystems). Relative CHIKV copy numbers of left and right joints and muscles were averaged for each animal. For the mouse studies, primer and probe sets, as previously described [40], were used in a one-step reverse transcription (RT)-qPCR assay. Perfused mouse ankles were homogenized in 1 ml of RLT buffer plus approximately 200 μl of silica beads using a MagNA Lyser instrument (Roche). RNA was extracted using the RNeasy mini kit (Qiagen) following manufacturer’s instructions and eluted in 50 μl of RNase-free water. A one-step qRT-PCR assay (Applied Biosystems) was performed with 2 μl of RNA using a 7500 Fast real-time PCR machine (Applied Biosystems). A standard curve was generated with serial dilutions of RNA extracted from virus stocks to determine CHIKV FFU equivalents. RNA was quantified using a Nanodrop spectrophotometer and total μg of RNA was determined.

### CHIKV isolation from tissues

NHP tissues were homogenized in 1 ml of cell culture medium plus approximately 250 μl of SiLiBeads using a bead beater (Precellys 24 homogenizer) (Bertin Technologies), and cellular debris was pelleted by centrifugation (5,000 × *g* for 2 min). Clarified samples were sterile-filtered (0.45 μm filter), and a 400 μl sample of the clarified lysate was applied to a T25 flask of C6/36 cells for three days. Supernatant titers from these cultures were determined by limiting dilution plaque assays on Vero cells. Mouse tissues were titered by focus forming assay. Perfused ankles were homogenized in 1 ml of cell culture medium plus approximately 200 μl of silica beads using the MagNA Lyser. Clarified homogenate was serially diluted and added to a confluent Vero cells cultured in a 96-well plate. After 2 h, tissue inoculum was removed and cells were overlaid with a carboxy methylcellulose-containing medium. Cells were fixed 18 h later and processed as described above.

### Phenotypic analysis of PBMCs

PBMCs were analyzed by flow cytometry for cellular differentiation markers as well as Ki67 (marker of active proliferation) or CD169 (activation marker for myeloid lineage cells and natural killer (NK) cells) as previously described [39]. T cell panel analysis was performed at all time points, and dendritic cell (DC)/monocyte/macrophage/NK cell panel analysis was performed on PBMCs from 0–5 dpi. For T cell analysis, PBMCs were stained with fluorophore-conjugated antibodies directed against CD4, CD8β, CD28, CD95, CD127 and Ki67 (Biolegend). For DC/monocyte/macrophage/NK analysis, PBMCs were stained with fluorophore-conjugated antibodies against CD3, CD20, CD14, HLA-DR, CD11c, CD123, CD16, CD8, and CD169 (Biolegend). The T cell and DC/monocyte/macrophage/NK cell gating strategy is depicted in **S2 Fig** and **S3 Fig**, respectively. Stained samples were read using an LSRII instrument (BD Pharminogen) and the data were analyzed by FlowJo (TreeStar).

### CHIKV-specific T cell responses

Monkey gamma interferon (IFNγ) enzyme linked immunospot assays (ELISpotPlus; Mabtech) were used to quantify CHIKV-specific T cell responses in peripheral blood of infected NHP at 7 dpi. Assays were performed according to the manufacturer’s instructions. Briefly, ELISpot plates were washed with PBS and blocked with RPMI containing 10% FBS, penicillin/streptomycin, and glutamine. Approximately, 1.5 x 10^5^ rhesus monkey PBMCs were stimulated with: (1) 1 μl DMSO-negative control; (2) overlapping CHIKV peptide pools (10 μg/well; ThermoScientific [39]) of nsP1, nsP2, nsP3, nsP4, capsid, E3, E2, 6K, or E1; (3) 10 μg of inactivated CHIKV; or (4) 1 μl each of phorbol 12-myristate 13-acetate (PMA; 40 μM stock) and ionomycin (7 mM stock)-positive control. The inactivated whole virus was prepared using 3 mM binary ethylenimine (BEI) treatment overnight followed by clarification through a 20% sucrose cushion at 22,000 rpm (68,128 x g) for 3 hrs; BEI treatment is an established inactivation technique for a number of viruses [41–43]. Successful inactivation of the virus was confirmed by plaque assay. The ELISpot plates were incubated at 37°C for 18 h and then washed with PBS. Detection of IFNγ was performed by incubating the plates for 2 h with a biotin-conjugated anti-IFNγ antibody (7-B6-1, Mabtech) diluted in PBS with 0.5% FCS. Following this incubation, the plates were washed with PBS and then incubated with streptavidin-alkaline phosphatase in PBS plus 0.5% FBS for 1 h. Plates were washed and then developed with filtered 5-bromo-4-chloro-3-indolyl-phosphate/nitro blue tetrazolium (BCIP/NBT) substrate solution; flushing the wells with water stopped this reaction. The plates were air-dried and scanned using the AID EliSpot Reader Classic (AID). The average spot forming units per stimulus were calculated and graphed using Graph Pad Prism v6 software.

### Serum cytokine assays

Monkey cytokine assays were performed using a Cytokine Monkey Magnetic 29-plex Panel for Luminex Platform Kit (Invitrogen) according to the the manufacturer’s instructions using a 7-point standard curve. Following a 2 h incubation with monkey plasma, beads were washed twice and then labeled with biotinylated detector antibody for 1 h. Following additional washes, beads were incubated with streptavidin conjugated to R-Phycoerythrin for 30 min and washed. Cytokines were identified and quantified using a Luminex 200 Detection system (Luminex).

### Histological analysis

Rhesus macaque joint tissue fixed in 10% buffered formalin was routinely processed, embedded in paraffin, sectioned at 5 microns, and stained with hematoxylin and eosin. Sections of twelve joints were examined by two pathologists (ADL; LMAC) blinded to group assignment. A semiquantitative scoring system was developed by assessing inflammation which occurred in periarticular fat, tendon, connective tissue and/or skeletal muscle. Each joint was scored individiually and then cumulative socres were calculated for each animal.

### Statistical analysis

All statistical analysis was performed in Prism v6 software (GraphPad Software, Inc). EC_50_ values were determined by non-linear regression. For plasma viral load and tissue viral burden from monkeys, data was log-transformed and Dunnett’s multiple comparison test was performed. For mouse viral burden experiments, data was log-transformed, and the Mann-Whitney test was used. For cytokine and chemokine analysis, a Sidak’s multiple comparison test was used to determine significance.

## Results

### SVIR001 neutralizes infection in cell culture and in mice

**Fig 1:**
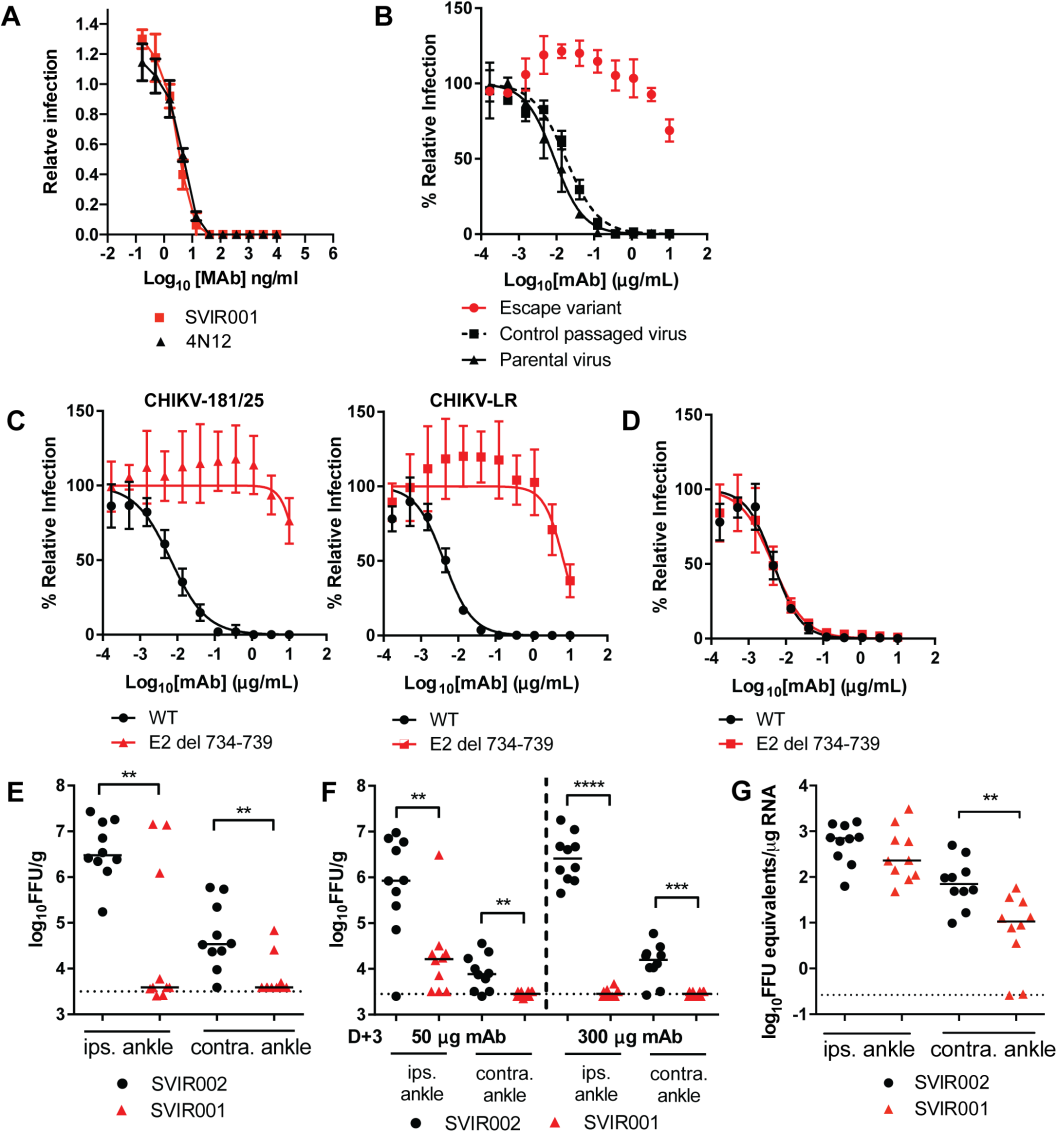
Characterization of neutralization, escape, and therapeutic efficacy of SVIR001 in mice. **(A)** SVIR001 and parent 4N12 mAbs were evaluated by neutralization assay in Vero cells. Virus was pre-incubated with indicated concentrations of mAb for 1 h and used to inoculate Vero cells. Data are reported as the relative infection normalized to a no mAb control. Results are representative of one of three independent experiments performed in duplicate. **(B)** The escape variant virus, which was generated by serial passage in the presence of SVIR001, was subjected to neutralization with SVIR001 and compared to a virus passaged in the absence of SVIR001 (control passaged virus). Results are representative of one of three independent experiments performed in duplicate. **(C-D)** Confirmation of SVIR001 escape phenotype with engineered six nucleotide deletion (E2 del 734–739). WT or deletion CHIKV-181/25 or CHIKV-LR viruses were incubated with indicated mAb for 1 h. Virus-mAb mixture was added to Vero cells. Data was normalized to a no mAb control. Each graph is two independent experiments done in triplicate and the mean ± SD are shown. **(E-G)** WT mice were inoculated subcutaneously with 10^3^ FFU of CHIKV-LR in the footpad and treated with 50 μg **(E-F)** or 300 μg **(F-G)** of anti-CHIKV mAb SVIR001 or control mAb SVIR002 at 1 **(E)**, 3 **(F)**, or 3 and 10 **(G)** dpi. At 3 **(E)**, 5 **(F)**, or 28 **(G)** dpi, virus was quantified by infectious focus **(E-F)** or qRT-PCR **(G)** assays from the ipsilateral and contralateral ankle to determine therapeutic efficacy. The median value is shown with the limit of detection indicated by the dotted line. Statistics were calculated on log-transformed data using the Mann-Whitney test (**, *P* < 0.01, ***, *P <* 0.001, ****, *P <* 0.0001). Each data point represents an individual animal. The data **(E-G)** were pooled from 2 independent experiments.

Our previous study demonstrated that combination therapy in NHPs with two mouse mAbs directed against CHIKV E1 and E2 proteins reduced spread but had limited efficacy at reducing viral burden in tissues that were infected prior to treatment (*i*.*e*., arm and finger joints and muscle) [38]. Our goal is to develop therapies against CHIKV that could be used during infection to reduce both viral load in tissues and clinical disease. In the current studies, we modified a previously described human mAb (4N12) that demonstrated therapeutic efficacy in immunocompromised mice [28]. We generated a synthetic human IgG1 kappa antibody, SVIR001, that had identical complementarity determining region amino acid sequences to mAb 4N12. To confirm that SVIR001 was functionally equivalent to 4N12, we compared their neutralization capacities directly. Both mAbs showed similar inhibitory activity to CHIKV-181/25 in Vero cells (**Fig 1A**); the concentration required to inhibit 50% of infection (EC_50_) was similar (SVIR001 EC_50_ = 4.1 ng/ml (95%CI 3.2–5.1); 4N12 EC_50_ = 5.0 ng/ml (95%CI: 4.3–5.9)), with no statistically significant differences.

4N12 was previously shown to bind E2, and mutagenesis of the arch domain of E2 at position D250A prevented 4N12 binding [28]. To determine if the targeting epitope of SVIR001 resembles that of 4N12, CHIKV-181/25 was passaged serially six times in the presence of 10 μg/ml of SVIR001 to obtain a virus that was resistant to SVIR001 inhibition. The escape variant was no longer sensitive to neutralization by SVIR001, whereas infection with the parent virus was neutralized by SVIR001 (**Fig 1B**). Sequencing of the gene encoding E2 of the SVIR001 escape variant virus revealed a six nucleotide deletion in E2 (nucleotides 734–739). The deletion resulted in substitution of amino acids 245–247 with a single lysine residue (NAE → K) (**S1A Fig**). This deletion is adjacent to Asp250, which is important for 4N12 binding to E2, suggesting that SVIR001 has a similar antigen binding site to 4N12. This six nucleotide deletion has not been noted to occur naturally, as determined by an alignment of 300 complete genome sequences of CHIKV (http://www.viprbrc.org). To confirm the escape mutation, the deletion was engineered into the CHIKV-181/25 and CHIKV-LR infectious clones (E2 del 734–739) (**S1B and S1C Fig)**. The 181/25 and LR E2 del 734–739 viruses were resistant to SVIR001, whereas the WT viruses were neutralized by SVIR001 (**Fig 1C**). The LR E2 del 734–739 and WT viruses however, were neutralized equivalently by a different human anti-CHIKV mAb (1H12), which bound domain A, B, and arch domain of E2 (**Fig 1D**) [28]. This result suggested that the six-nucleotide deletion did not cause widespread effects on E2 folding, and that possible changes in particle-to-plaque forming unit (PFU) ratio between the WT and mutant virus did not influence neutralization efficiency. These data suggest that SVIR001 and 4N12 bind to the same region of E2 and possess similar neutralizing profiles *in vitro*.

Our prior studies suggested that the parent mAb 4N12 was effective at mitigating CHIKV-induced morbidity and mortality in infected *Ifnar-/-* mice [28]. To assess the *in vivo* therapeutic efficacy of SVIR001, we used a 4 week-old WT mouse model of CHIKV infection in which animals are inoculated subcutaneously in the foot and virus replicates preferentially in musculoskeletal tissues [35], as seen in humans. In initial therapeutic studies, we treated CHIKV-infected WT mice one day post-inoculation (1 dpi) with 50 μg (~3.3 mg/kg) of SVIR001 or control SVIR002 via an intraperitoneal route and measured infectious viral burden in the ipsilateral and contralateral ankles at 3 dpi. Treatment with SVIR001 resulted in significantly lower viral titers in both tissues (**Fig 1E**). We extended the window of treatment and administered a single dose of either 50 or 300 μg (3.3 to 20 mg/kg) at 3 dpi and analyzed viral burden at 5 dpi. SVIR001 treatment significantly reduced viral load in the ipsilateral and contralateral ankles at 5 dpi (**Fig 1F**). Moreover, treatment with 300 μg (20 mg/kg) of SVIR001 at 3 and 10 dpi also resulted in lower levels of CHIKV RNA in the contralateral ankle in the chronic phase, at 28 dpi (**Fig 1G**). Collectively, the protective activity of SVIR001 in WT mice demonstrates its feasibility as a post-exposure therapeutic for CHIKV [28].

### SVIR001 reduces viral burden in CHIKV-infected rhesus macaques

To further characterize the efficacy of SVIR001, we used a rhesus macaque model of CHIKV infection [38, 39]. Animals were inoculated subcutaneously with a total of 10^7^ PFU of CHIKV-LR injected into ten sites on both arms. Following infection, animals displayed erythema and swelling of arm joints (especially the wrist) beginning at 1 to 2 dpi that lasted until 5 dpi. The affected joints were warm to the touch compared to unaffected areas, indicating active inflammation. Most animals developed a maculopapular rash, and upper extremity edema that was consistent with previous experiments in NHPs [39, 44]. This effect occurred irrespective of treatment group and the degree of the rash and swelling appeared different for each individual monkey. At day 1 and 3 of infection, animals received CHIKV mAb SVIR001 (5 or 15 mg/kg) or isotype control antibody SVIR002 (15 mg/kg) diluted in saline by intravenous infusion (**Table 1**).

**Fig 2:**
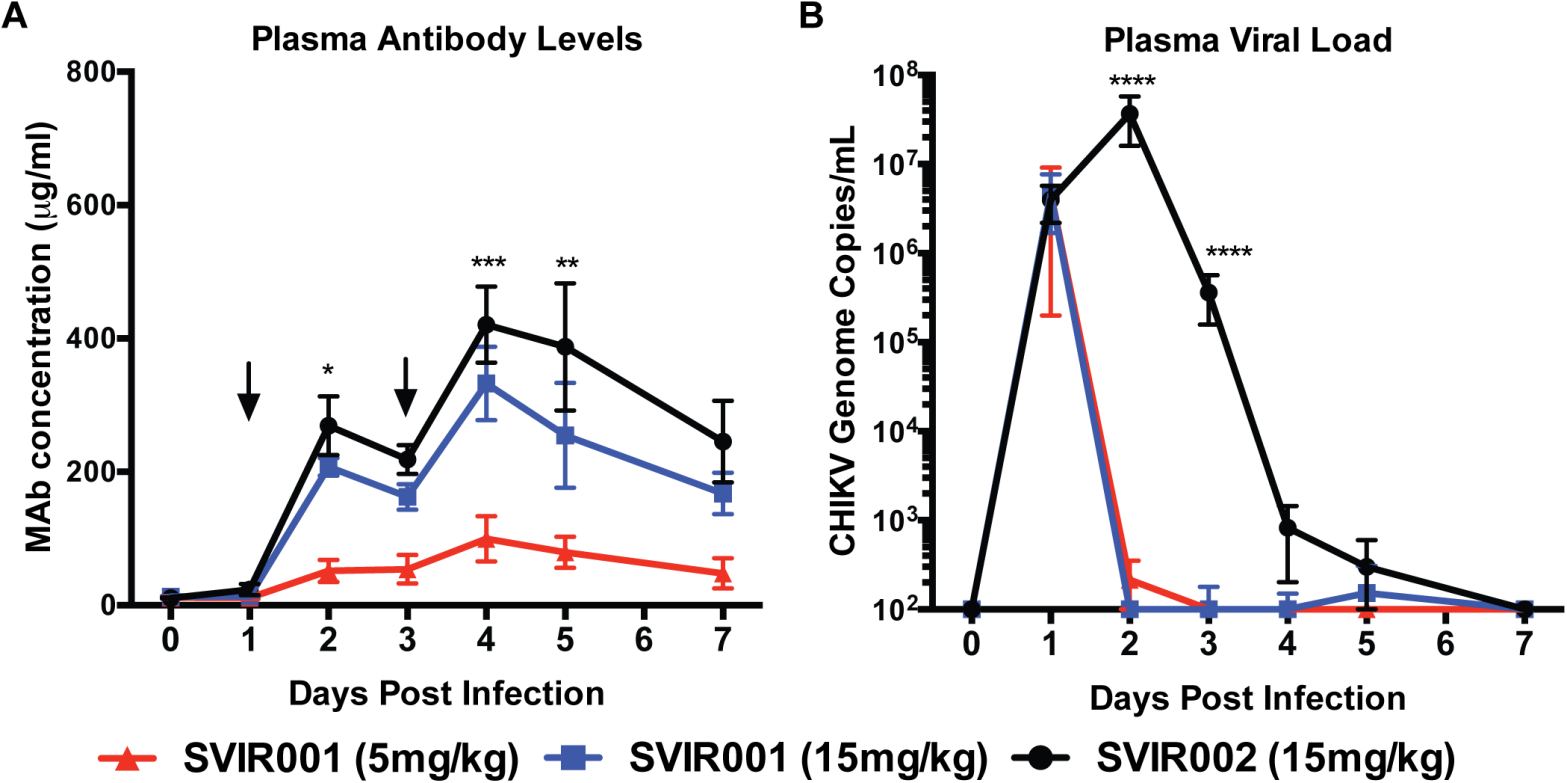
Plasma antibody levels and viral load following CHIKV mAb therapy. Rhesus macaques were inoculated subcutaneously in both arms with 1 x 10^7^ PFU of CHIKV LR. On day 1 and 3 after infection, rhesus macaques were administered 5 or 15 mg/kg SVIR001 (human anti-CHIKV mAb) or 15 mg/kg SVIR002 (human anti-lysozyme mAb), n = 4/group. Blood was collected on 0, 1, 2, 3, 4, 5, and 7 dpi. **(A)** Human mAb concentration in the plasma was measured by ELISA with lysozyme or CHIKV virions, and mAb concentration was calculated using a standard curve. Statistical significance was calculated using Tukey’s multiple comparison test (n = 4; ***, *P* < 0.0005, **, *P* < 0.01, *, *P* < 0.05). **(B)** Virus was quantified from plasma by qRT-PCR. Statistically significant differences are reported on the log-transformed data using Dunnett’s multiple comparison test (n = 4; ****, *P* < 0.0005).

Rhesus macaque infection with CHIKV results in viremia that is detectable at day 1 and lasts for about 3 to 4 days [38, 39]. To determine the effects of SVIR001 on viremia, blood was collected on 0, 1, 2, 3, 4, 5, and 7 dpi. The blood drawn on day 1 and 3 after infection occurred prior to antibody administration. Human antibody levels were measured at all time points by ELISA; all animals were positive for human anti-CHIKV (SVIR001) or anti-lysozyme antibodies (SVIR002) after the first i.v. treatment (**Fig 2A**). Antibody levels peaked at 2 and 4 dpi after the first and second antibody administration, respectively. The SVIR001 and SVIR002 15 mg/kg dose groups had similar peak plasma human antibody levels at 2 dpi (208 ± 26 μg/ml and 269 ± 88 μg/ml, respectively) and at 4 dpi (333 ± 110 μg/ml and 420 ±114 μg/ml, respectively). As expected, the group receiving 5 mg/kg SVIR001 had lower plasma human antibody levels at 2 and 4 dpi compared to the 15 mg/kg groups (52 ± 33 μg/ml and 100 ± 68 μg/ml, respectively). Plasma viral load in the presence of the control antibody SVIR002 was high at 1 dpi and peaked at 2 dpi (**Fig 2B**). The levels began to drop at 3 dpi with little residual virus detected at 5 and 7 dpi. Plasma from the animals treated with SVIR001 had high levels of CHIKV at 1 dpi, and levels dropped to an undetectable range at 2 dpi following treatment and remained undetectable through day 7. Only one SVIR001-treated animal had any detectable virus at 5 dpi, and the level was near the detection limit of the assay.

**Fig 3:**
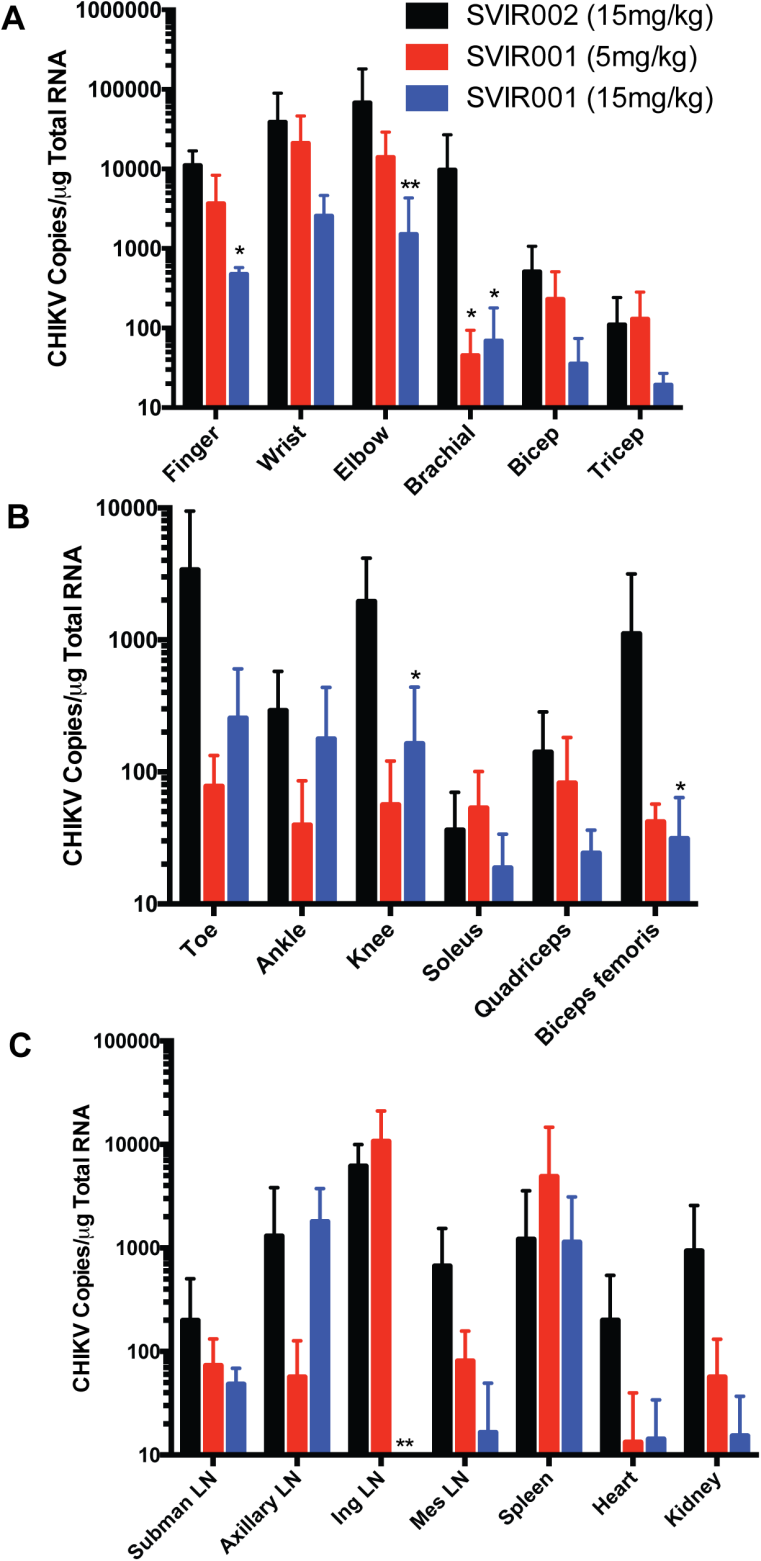
Tissue viral load following CHIKV mAb therapy. Animals were euthanized at day 7 post-infection, and viral RNA was isolated from tissues and quantified by qRT-PCR. The viral load in **(A)** arm joints and muscles, **(B)** leg joints and muscles, and **(C)** lymphoid tissues, heart and kidney are reported. Statistical significance was determined on the log-transformed data using Dunnett’s multiple comparison test, and multiplicity-adjusted *P* values are reported (n = 4; ** *P* < 0.005, * *P* < 0.05).

In NHP, CHIKV rapidly disseminates (within 2 days) from the initial site of infection, and is present in many tissues at 7 dpi, including joint and musculoskeletal tissues, lymph nodes, spleen, heart, lung, and kidney [38]. To demonstrate the efficacy of the mAb treatment on virus infection and dissemination, animals were euthanized at 7 dpi for tissue viral load analysis. In contrast to control-treated animals, where infectious virus was isolated from the tissue of at least one joint of all animals, infectious virus was not recovered from any tissue in animals receiving anti-CHIKV mAb (**Table 1**). Viral RNA levels in tissues were reduced in the SVIR001 anti-CHIKV mAb treated animals compared to controls in the arm joint tissues and muscles (**Fig 3A**). The reduction of viral RNA in the arms following SVI001 therapy was superior to previous results with a combination of anti-CHIKV mAbs, which failed to lower viral loads in the arms of infected macaques [38]. Animals treated with 15 mg/kg SVIR001 had reduced viral load in finger joints (1.1 x 10^4^ ± 5.8 x 10^3^ vs. 4.8 x 10^2^ ± 1.0 x 10^2^ copies/μg; *P* < 0.04) and elbow joints (6.8 x 10^4^ ± 1.1 x 10^5^ vs. 1.5 x 10^3^ ± 2.8 x 10^3^ copies/μg; *P* < 0.002). CHIKV infection in the brachial muscle was reduced in both SVIR001 treatment groups (9.7 x 10^3^ ± 1.7 x 10^4^ vs. 45 ± 49 and 69 ± 1.1 x 10^2^ copies/μg, respectively; *P* < 0.03). Infection also was reduced at distant musculoskeletal sites by the anti-CHIKV mAb compared to control-treated animals. Viral load was decreased in the 15 mg/kg SVIR001 treatment group in the knee (2.0 x 10^3^ ± 2.2 x 10^3^ vs. 1.6 x 10^2^ ±2.8 x 10^2^ copies/μg; *P* < 0.03) and biceps femoris muscle (1.1 x 10^3^ ± 2.0 x 10^3^ vs. 31 ± 33 copies/μg; *P* < 0.03) (**Fig 3B**). We also observed a reduction of viral load in submandibular lymph nodes, inguinal lymph nodes, mesenteric lymph nodes, heart, and kidney (**Fig 3C**). In some tissues there was an SVIR001 concentration-dependent decrease in viral loads with a greater impact seen with the higher 15 mg/kg dose. This effect was most evident in the joint tissues of the arms. Similarly, viral loads in the inguinal lymph nodes were undetected with high dose treatment of SVIR001, which is consistent with the overall reduction of infection in the legs (6.2 x 10^3^ ± 3.8 x 10^3^ vs. 3.3 ± 6.5 copies/μg; *P* < 0.004). No effect of treatment on CHIKV RNA levels was observed in the spleen, as was seen previously [38]. These results establish the efficacy of SVIR001 therapy in reducing tissue viral load at the site of infection and spread to distant sites.

### SVIR001 inhibited CHIKV-associated inflammation

**Fig 4:**
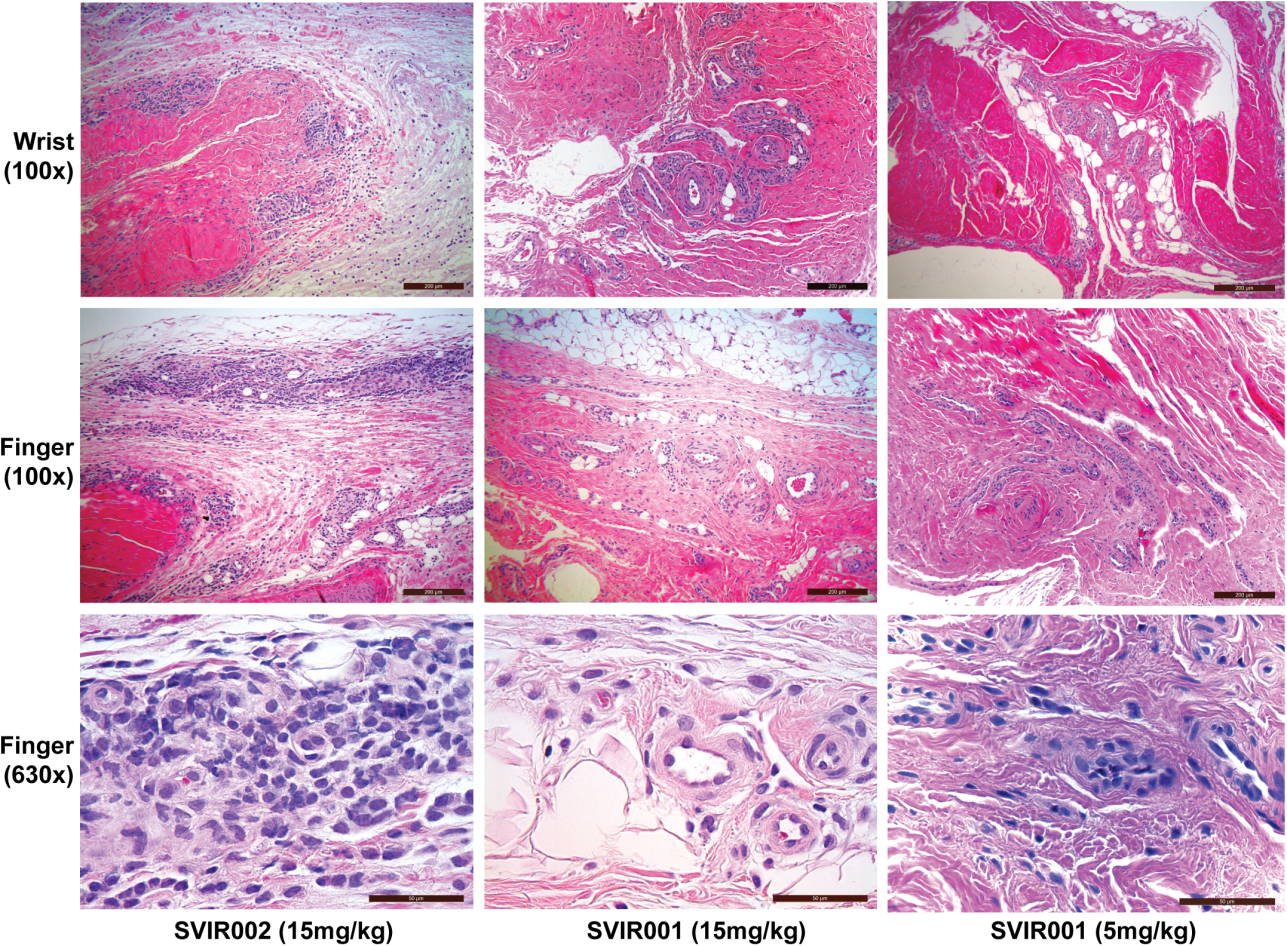
Histological images of joint-associated tissue from CHIKV-infected animals at 7 dpi. At 7 dpi, joint-associated tissue from the wrist and finger of each animal were fixed, paraffin embedded, sectioned, and stained with hematoxylin and eosin. Shown are representative images of stained sections from joint-associated soft tissue of four animals treated with control antibody SVIR002 or CHIKV mAb SVIR001. In the SVIR002 treated animals, there was abundant inflammation surrounding multiple vessels. In the SVIR001 treated animals (5 or 15 mg/kg dose), there was limited or no perivascular inflammation.

To evaluate the impact of anti-CHIKV mAb treatment on cellular infiltration into the joints, we analyzed tissue sections from animals in each treatment group at 7 dpi. Sections consisted of the soft tissue from the finger, wrist, elbow, toe, ankle, and knee joints bilaterally. The animals treated with the control mAb SVIR002 had a higher average cumulative score (average score 10.75) with more inflammation in affected joints and higher numbers of joints affected within individuals (**Table 2, S3 Table**). Sections from animals treated with 5 mg/kg (average score 3.25) and 15 mg/kg (average score 4.75) of SVIR001 had less inflammation within affected joints and fewer joints affected (**Fig 4**). Collectively, these results suggest that in rhesus macaques, SVIR001 therapy reduced viral load in the periphery and thereby prevented or diminshed acute inflammation in musculoskeletal tissues infected with CHIKV.

**Table 2:** Histological findings in joint tissues at 7 dpi.

mAb	SVIR002 (15 mg/kg)	SVIR001 (15 mg/kg)	SVIR001 (5 mg/kg)
**Right finger**	1.5	1	0.25
**Left finger**	1.5	0.75	0.25
**Right wrist**	0.5	0.25	0.25
**Left wrist**	0.75	1.25	1
**Right elbow**	0.25	0.25	0.75
**Left elbow**	1.5	0.75	0.25
**Right toe**	0.5	0	0
**Left toe**	0.75	0	0
**Right ankle**	0.5	0	0
**Left ankle**	0.75	0.25	0
**Right knee**	1.25	0	0.25
**Left knee**	1	0.25	0.25
**Total score**	**10.75**	**4.75**	**3.25**
**# joints affected**	**7.75**	**3.75**	**3**

Each joint was evaluated for the presence of perivascular infiltrates of lymphocytes, histiocytes and plasma cells in hematoxylin and eosin stained microscopic sections and assigned a score between 0–3. A score of 0 indicates no evidence of inflammation; 1 indicates few perivascular infiltrates forming one layer and affecting three vessels or less; 2 indicates perivascular infiltrates forming one to three layers and affecting more than three vessels; 3 indicates perivascular infiltrates forming four or more layers. The average score for each treatment group is reported (n = 4). See **S3 Table** for the scores for each animal.

**Fig 5:**
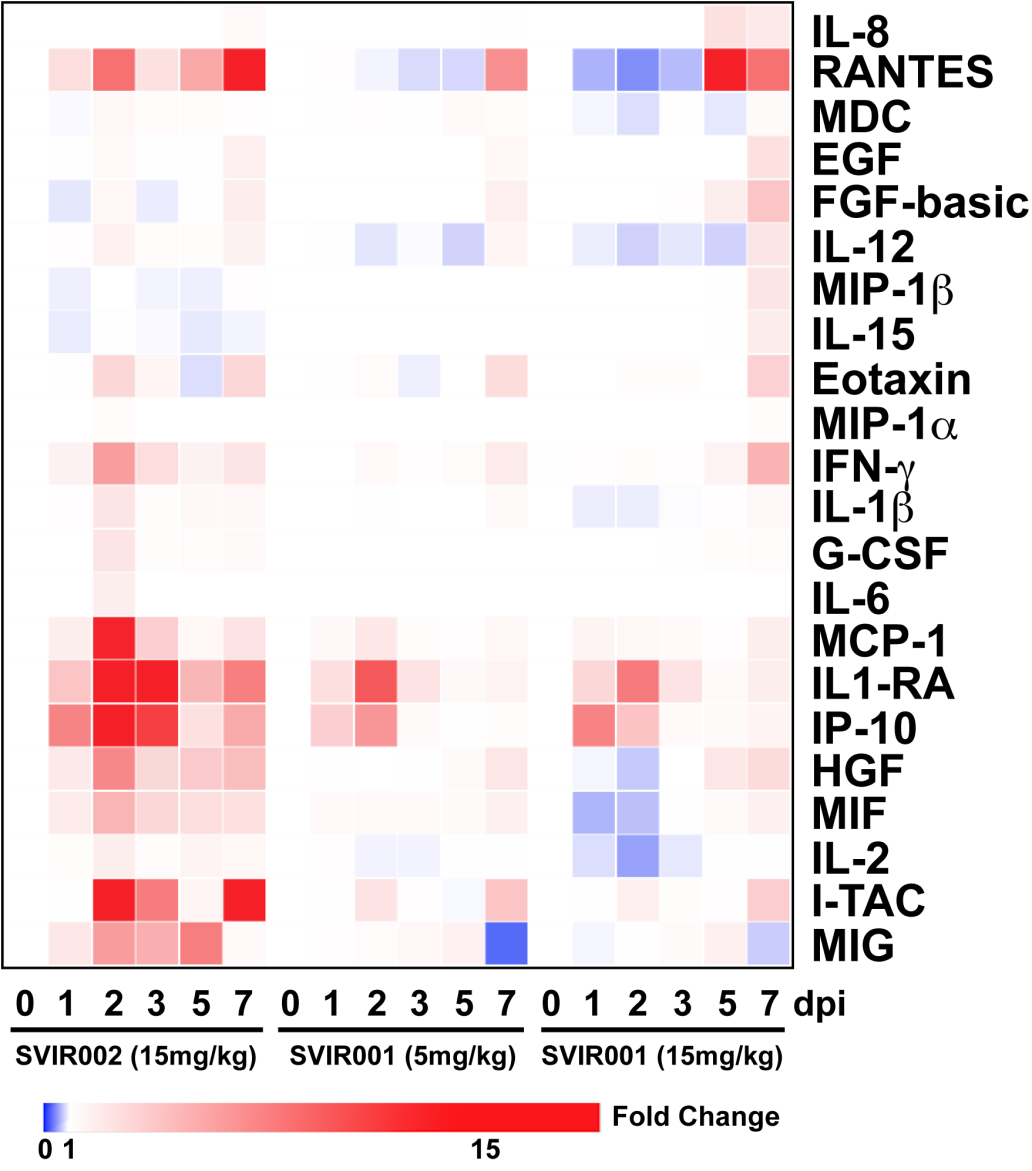
CHIKV mAb therapy reduced plasma cytokines and chemokine activation at 2 dpi. Heat map comparing average fold-change in plasma cytokine profiles for NHP treated inoculated with CHIKV and treated with control antibody SVIR002 or CHIKV mAb SVIR001. Clustering was performed using the Broad Institute’s webtool Morpheus. A 29-plex-cytokine magnetic bead assay was performed on plasma from rhesus macaques isolated at day 0, 1, 2, 3, 5, and 7 post-infection.

To understand the basis for reduced cellular inflammation conferred by SVIR001 treatment, we measured chemokine and cytokine levels in plasma over time on days 0, 1, 2, 3, 5, and 7 following CHIKV infection using a quantitative 29-plex cytokine magnetic bead assay. In the control antibody treatment group, there was a significant increase in plasma levels of IL-1β, G-CSF, IL-6, Eotaxin, MIP-1α, MCP-1, HGF, IFNγ, I-TAC, MIF, IL-1RA, IP-10, and MIG at 2 dpi (**Fig 5 and S3 Fig**), which coincides with peak viremia. However, plasma from the groups that received CHIKV mAb therapy did not show increased levels of these cytokines and chemokines at 2 dpi. In comparison, plasma levels of FGF-basic, IL-10, IL-12, RANTES, IL-17, GM-CSF, MIP-1β, IL-15, EGF, IL-5, VEGF, MDC, TNFα, IL-2, IL-4, and IL-8 were either undetectable or without differences between the treatment groups (**S3 Fig**). The decrease of plasma cytokine/chemokine induction at 2 dpi in the SVIR001-treated animals could account in part for the reduction in joint tissue cell infiltration and inflammation.

**Fig 6:**
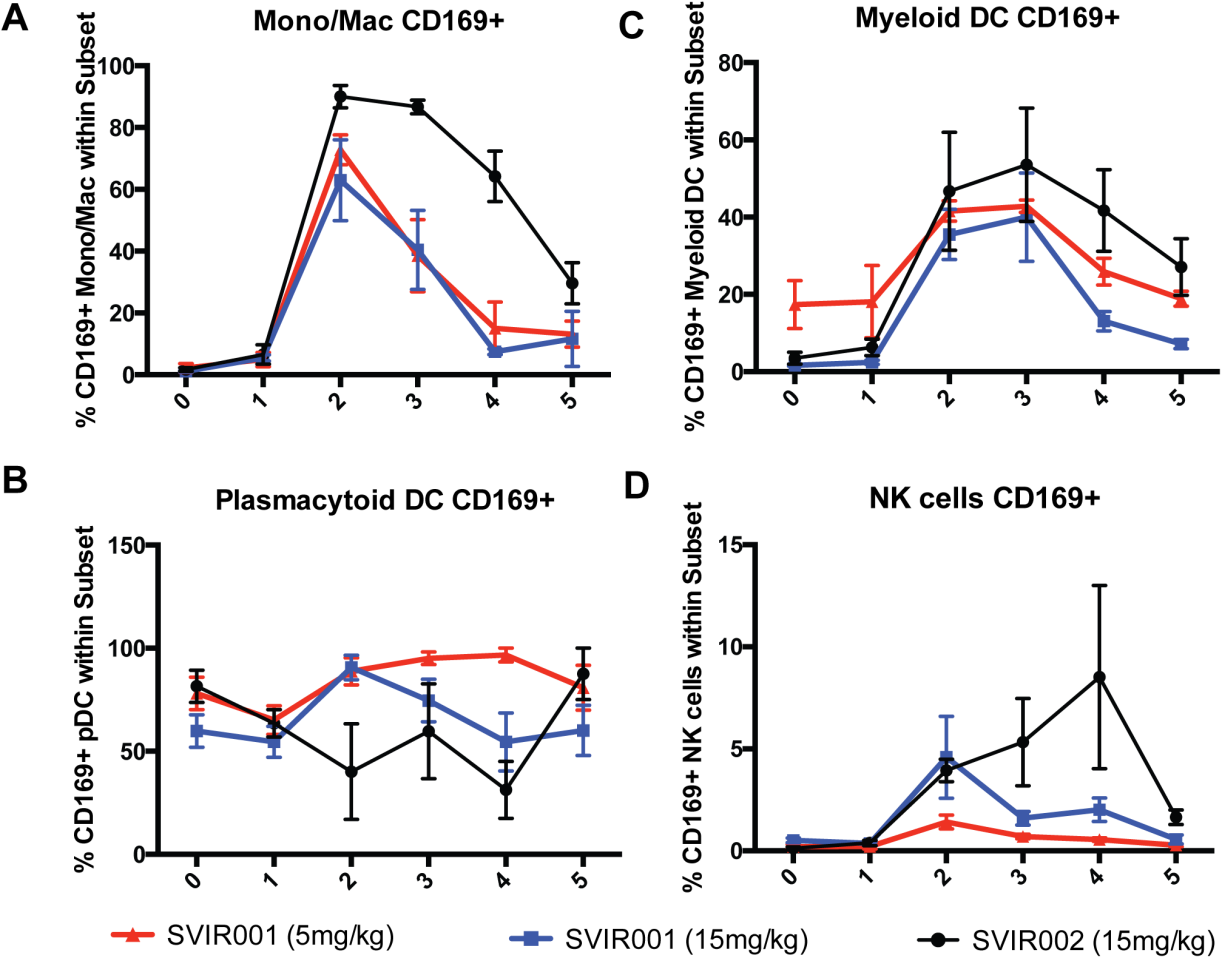
CHIKV mAb therapy reduced activation of peripheral blood monocytes/macrophages, Myeloid DCs, and NK cells. Total peripheral blood mononuclear cells from 0, 1, 2, 3, 4 and 5 dpi were stained with antibodies directed against HLA-DR, CD14, CD169, CD11c, CD20, CD3, CD8, and CD16 to assess changes in the activation of macrophage/monocyte, DC, and NK cell subsets. The percent of activated, CD169^+^ **(A)** monocyte/macrophages, **(B)** plasmacytoid DCs, **(C)** myeloid DCs, or **(D)** NK cells within the population are reported (n = 4).

We next assessed whether there were differences in levels of activated monocyte/macrophages, DCs, or NK cells in blood associated with SVIR001 treatment. PBMCs were stained with antibodies against the cell surface markers HLA-DR, CD14, CD169, CD11c, CD20, CD3, CD8, and CD16, and subsets were defined (**S2 Fig**). In all treatment groups, we observed an increase in activation of monocyte/macrophages and myeloid DCs as reflected by CD169^+^ staining peaking at 2–3 dpi followed by a steady decrease, returning to nearly baseline levels (**Fig 6A**). However, the number of activated monocytes/macrophages and mDCs was higher and endured longer in the control mAb-treated (SVIR002) compared to SVIR001-treated animals (**Fig 6A–6C**). Similarly, NK cell activation (CD169^+^ staining) was sustained in the control-treated compared to anti-CHIKV antibody-treated animals (**Fig 6D**). These results, together with decreased plasma levels of inflammatory chemokines and cytokines and decreased cellular infiltration into the joints of SVIR001 treated animals, suggest that SVIR001 therapy reduced viral burden and consequently inhibited cellular and soluble mediators of inflammation.

### SVIR001 treatment did not diminish anti-CHIKV adaptive immunity

**Fig 7:**
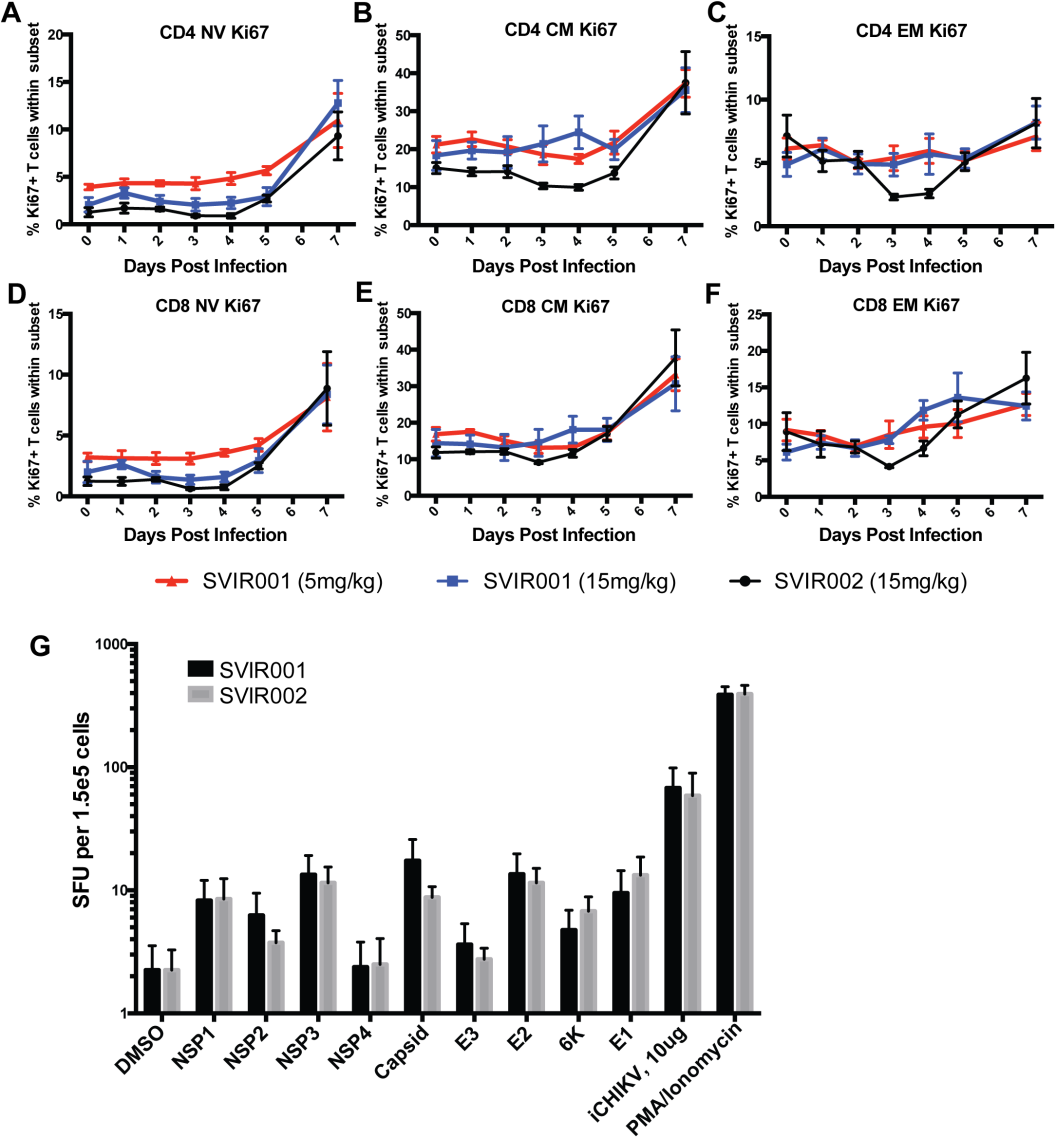
CHIKV mAb treatment did not cause significant changes in CD4^+^ or CD8^+^ T cell proliferation. Rhesus macaques were inoculated with CHIKV and treated with control antibody SVIR002 or CHIKV mAb SVIR001. Blood was drawn daily 0–7 dpi, and PBMCs were examined for proliferative responses of different **(A-C)** CD4^+^ and **(D-F)** CD8^+^ T cell subsets. T cell subsets were defined in **S2 Fig** as Naïve (NV), Central Memory (CM), and Effector Memory (EM). The Ki67^+^ proliferative status was plotted as a percentage of the total population. **(G)** IFNγ ELISpot analysis was performed on PBMCs from rhesus macaques at 7 dpi. PBMCs from animals treated with SVIR001 (5 mg/kg or 15 mg/kg) or SVIR002 (15 mg/kg) were stimulated with CHIKV peptide pools (10 μg/well), inactivated CHIKV (iCHIKV) (10 μg/well), or PMA/Ionomycin as a positive control. DMSO was used as a negative control to establish the baseline number of IFNγ-producing T cells for each animal. Spots were quantified on an AID ELISpot plate reader (n = 4/group).

We next determined whether anti-CHIKV mAb therapy altered lymphocyte mobilization and function. Flow cytometry was used to assess changes in frequency and phenotype of lymphocytes in blood during the course of infection. PBMCs were stained with CD4, CD8, CD95, and CD28 markers to differentiate naïve (NV), central memory (CM), and effector memory (EM) T cell subsets. Subsequently, the cells were stained with antibodies against Ki67 to determine the proportion of proliferating T cells. The gating strategies used to define the T cell subsets and phenotypes are shown in **S1 Fig**. Over the course of CHIKV infection, we observed an increase in proliferation of all CD4^+^ T cells subsets (NV, CM, and EM) with the maximal level observed at 7 dpi (**Fig 7A–7C**), as seen previously in NHPs [39]. We did not observe a significant difference in T cell phenotype or proliferation status between the SVIR001 and SVIR002 treated animals. Similar to the CD4^+^ T cell proliferative response, Ki67^+^CD8^+^ T cell subsets from both SVIR001- and SVIR002-treated animals increased throughout infection and the maximal level was observed at 7 dpi (**Fig 7D–7F**). Thus, treatment with SVIR001 did not negatively impact the generation of T cell proliferative responses despite reducing the viral antigen burden. We also did not observe differences in B cell proliferative responses between treatment groups (**S5 Fig**).

The functionality and frequency of the CHIKV-specific T-cell responses in the peripheral blood was measured using an IFNγ ELISpot. PBMCs isolated at 7 dpi were stimulated with overlapping peptide pools for the nine different CHIKV proteins [39], inactivated whole virus (iCHIKV), or PMA/ionomycin as a positive control. CHIKV-reactive T cells were present in animals from both the control mAb (SVIR002) and anti-CHIKV mAb (SVIR001) treatment groups (**Fig 7G**). We did not observe significant differences between the two groups, suggesting that the antibody treatment did not affect the breadth of the CHIKV-specific T cell response at 7 dpi. These results, along with the T cell proliferation profiles, suggest that SVIR001 treatment did not negatively impact the generation of anti-CHIKV specific T cell responses. Thus, an immunotherapeutic mAb against CHIKV can reduce viral loads and prevent CHIKV-induced joint disease without a detectable effect on the induction of antiviral adaptive immunity, which would prevent disease associated with a second infection.

## Discussion

CHIKV is a clinically relevant re-emerging alphavirus that causes chronic polyarthritis and even death in certain immunocompromised or elderly populations. In the current studies, we determined the efficacy of SVIR001, a fully human anti-CHIKV immunotherapeutic in mouse and rhesus macaque models. In WT mice, recombinant antibody substantially reduced levels of infectious virus from ankle tissues when given at 1 or 3 dpi, and reduced levels of persistent viral RNA in some joint tissues at 28 dpi. In NHPs, SVIR001 aborted CHIKV viremia, prevented its spread to distal joints and muscle, and reduced viral RNA loads in the hands and arms, the initial sites of infection. This feature of reducing viral loads in the joints of the arm was not observed in a previous study with two mouse anti-CHIKV mAbs (CHK-152 and CHK-166), suggesting that SVIR001 is more effective at clearing established infections [38]. SVIR001 also reduced CHIKV-induced joint disease, an effect that likely was due to reductions in viral burden and therefore reduced generalized inflammatory responses. However, this signature of reduced inflammation did not compromise induction of adaptive B or T cell immunity against the virus. These features make SVIR001 a candidate treatment for humans who are acutely infected by CHIKV or possibly those suffering from the long-term joint pain caused by persistent infection.

Acute CHIKV infection in rhesus macaques induces a viremia that lasts for approximately 3 to 4 days, with peak viral titers occurring around 2 to 3 dpi [38, 39, 45, 46]. Virus in plasma was not detected in animals receiving anti-CHIKV antibody therapy after the first treatment dose. Anti-CHIKV antibody likely neutralizes virus in the blood rapidly, which reduces dissemination to distant sites of infection including the joints and muscles of the legs. Consistent with this idea, tissue viral loads at 7 dpi were decreased markedly in animals treated with SVIR001. The reduction in tissue viral loads included those of the joint and muscle tissue from the arms, the site of infection, which contrasts with our previous findings with mouse CHK-152 and CHK-166 [38]. Although further studies are required, the marked improvement of this mAb therapy compared to our previous mAb combination therapy could be due to different specific mechanism of neutralization, a greater recognition of E2 on the surface of infected cells that augments effector-based clearance, or a longer relative half-life in plasma.

CHIKV disease is mediated by active replication in musculoskeletal tissues that promotes immune cell infiltration and production of inflammatory mediators, which causes synovitis, tenosynovitis, and bone effacement, and results in severe and acute joint pain [3, 13, 47, 48]. High CHIKV levels in human patients have been associated with increased severity of illness during acute infection [25, 49], and analogously, higher peak viremia in cynomolgus macaques was associated with worse outcomes including arthritis, meningoencephalitis, and death [45]. High tissue viral loads correlate with an increase in the duration of long-term arthralgia in patients [3]. Thus, antiviral therapies that reduce viral loads during the acute phase may limit joint and muscle disease and mitigate chronic CHIKV disease. Histological analysis of the joint tissues from the infected monkeys at 7 dpi revealed less severe acute disease in the SVIR001 treated compared to the control-treated animals. This observation included a reduction in the number of inflamed joints and a decreased severity of the disease. This finding was supported by a reduction in cellular infiltration and production of proinflammatory cytokines and chemokines in animals receiving SVIR001 therapy. Several of the cytokines that were diminished in plasma after SVIR001 treatment are elevated in human plasma during the symptomatic phase of CHIKV infection, including IFNγ, IP-10, IL-1β, MCP-1, MIG and IL-6 [50–53], with an increase in IL-1β and IL-6 being linked to more severe disease in humans [53].

SVIR001 and other effective anti-CHIKV therapeutics could serve as treatments for CHIKV infection. We previously showed the efficacy of 4N12 prophylaxis and acute therapy in *Ifnar-/-* mice [28]. We also demonstrated that treatment of mice and monkeys with immunotherapeutics during the viremic phase reduces virus dissemination to the distal joints and tissues [38]. An important, yet unanswered question, is what is the therapeutic window for antibody treatment against CHIKV? In humans the acute viremic phase following CHIKV infection lasts 4 to 12 days post symptom onset [14, 54, 55], making this window relatively narrow. The clinical utility of an antiviral that works solely by blocking viral dissemination may be limited due to delays in diagnosis of CHIKV infection during the viremic phase. Further studies are needed to determine whether SVIR001, alone or in combination with other antibodies or immunomodulatory agents, can reduce musculoskeletal disease, viral burden, or the transition to persistence when administered at later time points. The addition of a second antibody to the cocktail could limit the risk of emergence of resistance [38].

A theoretical concern of immunotherapeutic treatments for CHIKV and other viral infections is that by quickly eliminating antigen, they may interfere with the development of adaptive immunity and render the host susceptible to reinfection. However, we did not observe any differences in T or B cell proliferation or activation in response to infection when the therapy was initiated at 1 dpi. Similarly, we did not detect any differences in CHIKV-specific IFNγ-producing T cell responses at 7 dpi. These observations suggest that SVIR001 did not inhibit the induction of adaptive immunity and that long-term protection is most likely not compromised by an antiviral therapeutic antibody.

In summary, our results demonstrate that the anti-CHIKV mAb therapeutic SVIR001 reduced viremia and dissemination in infected animals and mitigated inflammation systemically and locally. Because SVIR001 promotes elimination of CHIKV from infected tissues, studies are warranted to determine its possible efficacy in preventing or treating viral persistence. Such studies will help to determine the potential clinical utility for prophylaxis or therapy for CHIKV disease.

## Supporting information

S1 Fig Sequence alignment of SVIR001 escape variant viruses. **(A)** Viral RNA was isolated from CHIKV-181/25 passaged in the presence of SVIR001, control passaged 181/25, or parental 181/25. cDNA was produced and sequenced to identify the escape mutation(s). The sequences were aligned to the CHIKV 181/25 infectious clone using MegAlign (DNAStar). **(B-C)** Results of mutagenesis. The six nucleotide deletion (del 734–739) was introduced into the CHIKV-181/25 **(B)** or CHIKV-LR **(C)** infectious clone. WT or deletion mutant viruses were generated in BHK21 cells. Viral RNA was isolated from stocks, cDNA was produced, and sequenced to confirm mutation. The sequences were aligned to the CHIKV 181/25 infectious clone **(B)** or CHIKV strain LR2006_OPY1 (accession number DQ443544) **(C)** using MegAlign (DNAStar). Deletion is highlighted in yellow.
(TIF)

S2 Fig T cell gating strategy. PBMCs were stained for surface levels of CD4, CD8β, CD95, CD28, CD127 and for intracellular levels of Ki67. The lymphocyte subset was identified and CD4^+^ and CD8^+^ T subsets are shown (top panel). Within the CD4^+^ and CD8^+^ T cell subsets, the naïve (CD28^+^CD95^-^), central memory (CD28^+^CD95^+^), and effector memory (CD28^-^CD95^+^) subsets are indicated. The percentage of proliferating (Ki67^+^) T cells within each subset was calculated.
(TIF)

S3 Fig Gating strategy for NK cells, macrophages, and DCs. PBMCs were stained with HLA-DR, CD14, CD11c, CD123, CD20, CD3, CD8, CD16, and CD169 to differentiate monocyte/macrophages, DCs, and NK cells using the following gating strategy: monocyte/macrophages (CD3^-^CD20^-^CD14^+^HLA-DR^+^), plasmacytoid DCs (CD3^-^CD20^-^CD14^-^HLA-DR^+^CD123^+^), myeloid DCs (CD3^-^CD20^-^CD14^-^HLA-DR^+^CD11c^+^), other DCs (CD3^-^CD20^-^CD14^-^HLA-DR^+^CD123^-^CD11c^-^), and NK cells (CD3^-^CD20^-^CD8^+^CD16^+^). The percentage of activated cells (CD169^+^) within each subset was calculated. The gating strategy and definition of the different cellular subsets are shown.
(TIF)

S4 Fig Plasma cytokines and chemokine analysis. Cytokine analysis from 29-plex-cytokine magnetic bead assay was performed on plasma from animals treated with SVIR001 or control mAb SVIR002. Cytokine analysis revealed changes in plasma cytokine levels of **(A)** IL-1β, **(B)** G-CSF, **(C)** IL-6, **(D)** eotaxin, **(E)** MIP-1α, **(F)** MCP-1, **(G)** HGF, **(H)** IFNγ, **(I)** I-TAC, **(J)** MIF, **(K)** IL-1RA, **(L)** IP-10, and **(M)** MIG. Differences were analyzed using Sidak’s multiple comparison tests, and adjusted *P* values are reported (n = 4; ****, *P* < 0.0001, ***, *P* < 0.0005, **, *P* < 0.01, *, *P* < 0.05). Individual animals are graphed. Plasma cytokine levels of **(N)** FGF-Basic, **(O)** IL-12, **(P)** RANTES, **(Q)** MIP-1β, **(R)** IL-15, **(S)** EGF, **(T)** MDC, **(U)** IL-2, and **(V)** IL-8 did not demonstrate any significant changes between treatment groups. IL-10, IL-17, GM-CSF, VEGF, TNFα, and IL-4 remained below the limit of detection and are not shown.
(TIF)

S5 Fig B cell proliferative responses were not affected by SVIR001 therapy. Total peripheral blood mononuclear cells were analyzed by flow cytometry for the presence of B cell proliferative responses following CHIKV infection in control and anti-CHIKV treated NHP. B cells were stained with antibodies directed against CD3, CD20, CD27, IgD and HLA-DR as well as Ki67 in order to identify proliferating (Ki67+) cells in naïve B cells, memory B cells and marginal zone like B cells. The percentage of actively proliferating cells within cell type was calculated using FlowJo software and the data was graphed in GraphPad Prism v6 software.
(TIF)

S1 Table Primers used for sequencing and amplifying the E2 and E1 genes of CHIKV-181/25. (TIFF)

S2 Table Oligonucleotide primers for mutagenesis of CHIKV infectious clones. (TIFF)

S3 Table Detailed histological findings reported per animal. H&E stained joint sections were scored as described in **Table 2**. Additional findings such as the presence of granulocytes or hemosiderin are indicated but were not used in the calculation of scores. * Granulocytes (eosinophils and/or neutrophils), # Hemosiderin
(TIFF)
