# Supplementary figures and images for: Therapeutic administration of a recombinant human monoclonal antibody reduces the severity of chikungunya virus disease in rhesus macaques
Source: PLoS Negl Trop Dis. 2017 Jun 19;11(6):e0005637. doi: 10.1371/journal.pntd.0005637 (PMC5491320; doi:10.1371/journal.pntd.0005637)

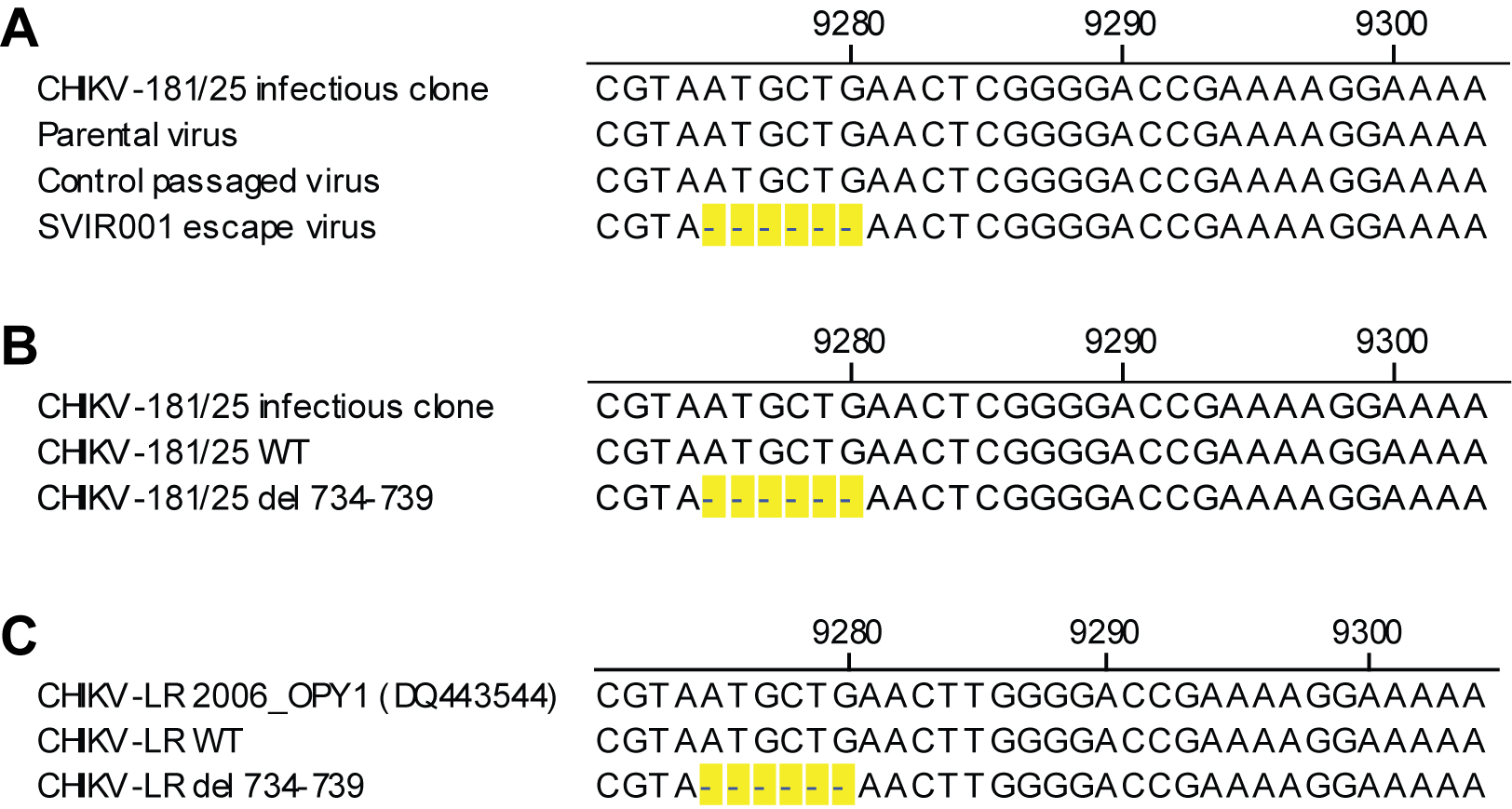

Supplement: S1 Fig — (A) Viral RNA was isolated from CHIKV-181/25 passaged in the presence of SVIR001, control passaged 181/25, or parental 181/25. cDNA was produced and sequenced to identify the escape mutation(s). The sequences were aligned to the CHIKV 181/25 infectious clone using MegAlign (DNAStar). (B-C) Results of mutagenesis. The six nucleotide deletion (del 734–739) was introduced into the CHIKV-181/25 (B) or CHIKV-LR (C) infectious clone. WT or deletion mutant viruses were generated in BHK21 cells. Viral RNA was isolated from stocks, cDNA was produced, and sequenced to confirm mutation. The sequences were aligned to the CHIKV 181/25 infectious clone (B) or CHIKV strain LR2006_OPY1 (accession number DQ443544) (C) using MegAlign (DNAStar). Deletion is highlighted in yellow. (TIF) [file pntd.0005637.s001.tif]

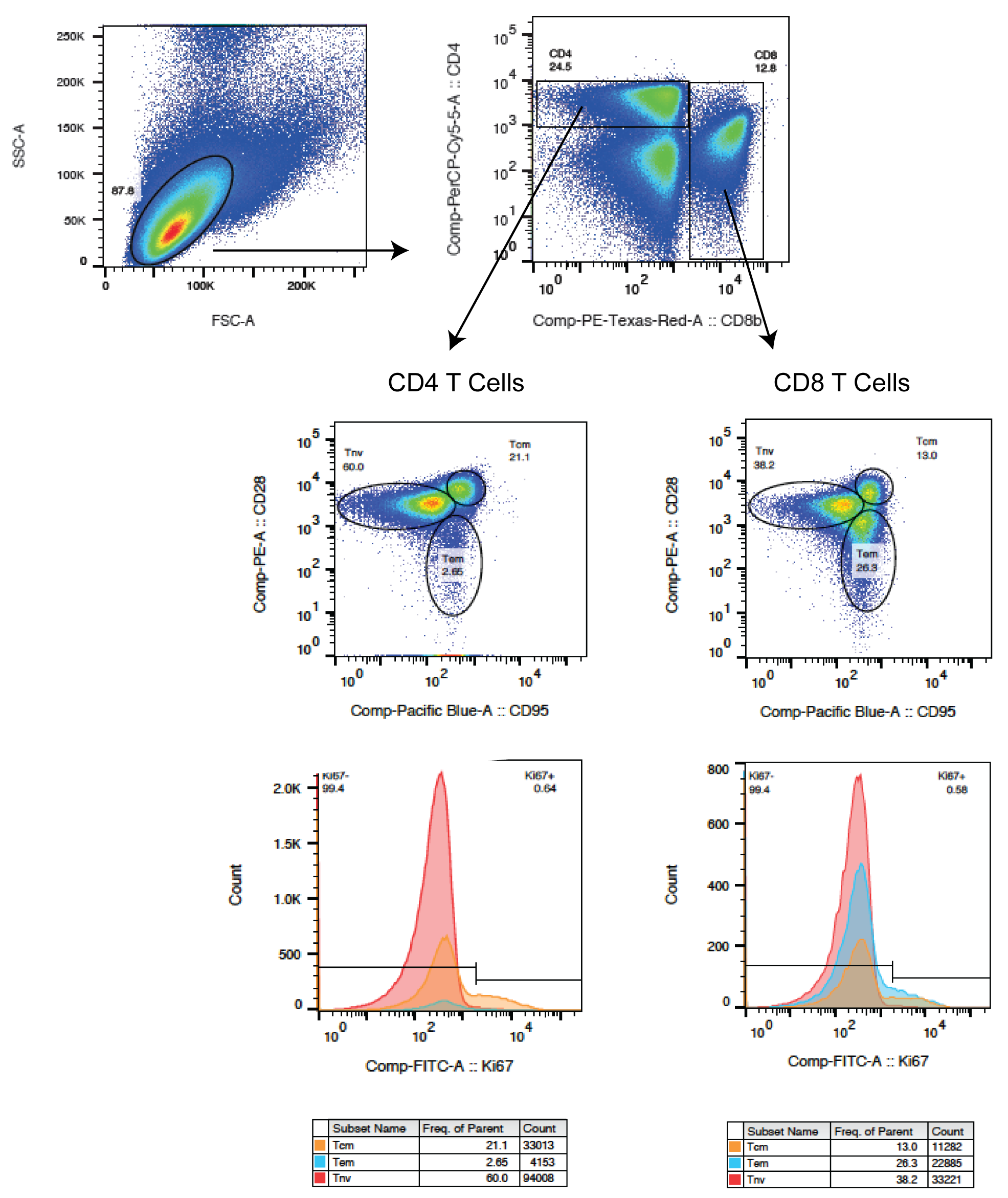

Supplement: S2 Fig — PBMCs were stained for surface levels of CD4, CD8β, CD95, CD28, CD127 and for intracellular levels of Ki67. The lymphocyte subset was identified and CD4+ and CD8+ T subsets are shown (top panel). Within the CD4+ and CD8+ T cell subsets, the naïve (CD28+CD95-), central memory (CD28+CD95+), and effector memory (CD28-CD95+) subsets are indicated. The percentage of proliferating (Ki67+) T cells within each subset was calculated. (TIF) [file pntd.0005637.s002.tif]

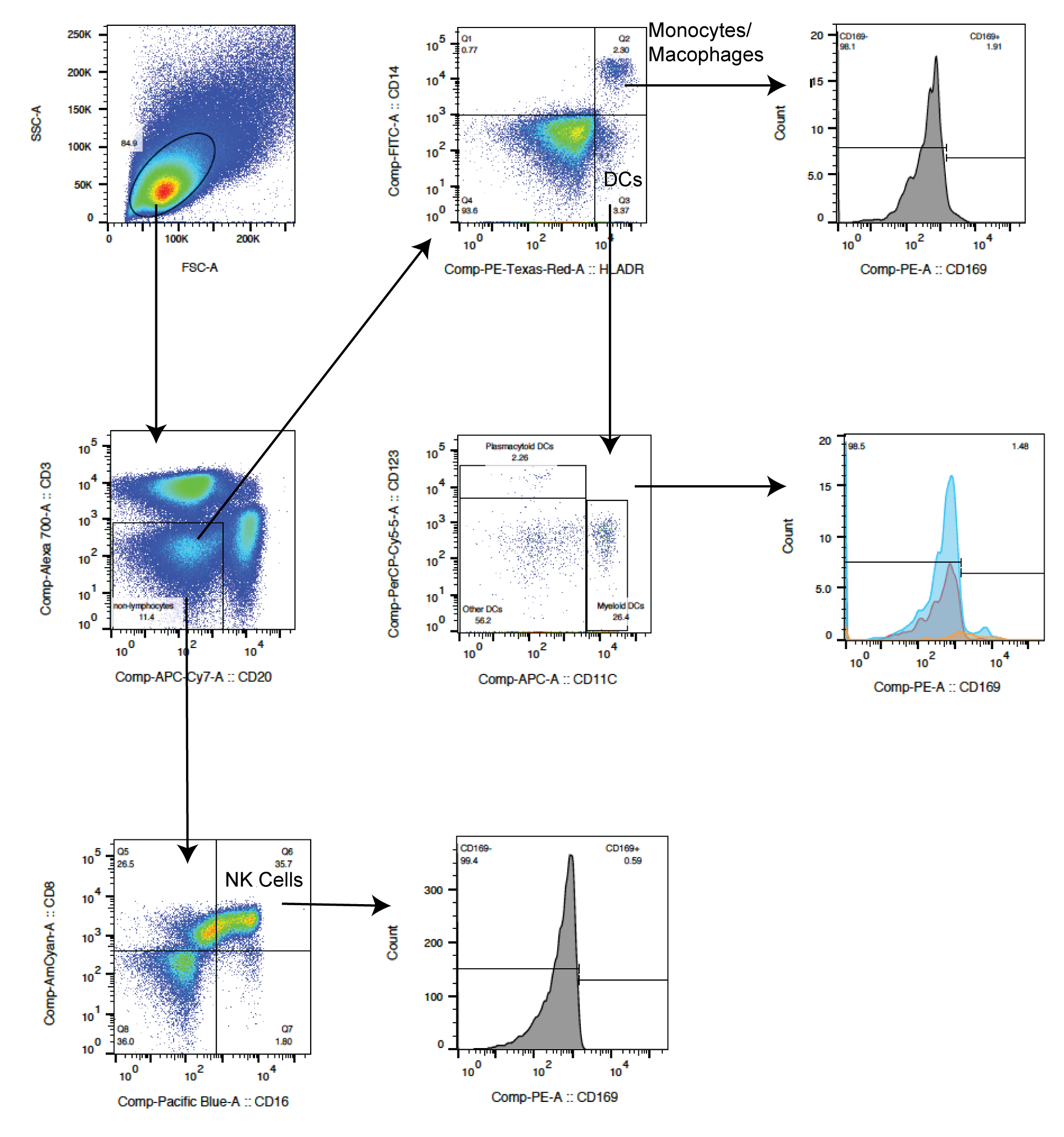

Supplement: S3 Fig — PBMCs were stained with HLA-DR, CD14, CD11c, CD123, CD20, CD3, CD8, CD16, and CD169 to differentiate monocyte/macrophages, DCs, and NK cells using the following gating strategy: monocyte/macrophages (CD3-CD20-CD14+HLA-DR+), plasmacytoid DCs (CD3-CD20-CD14-HLA-DR+CD123+), myeloid DCs (CD3-CD20-CD14-HLA-DR+CD11c+), other DCs (CD3-CD20-CD14-HLA-DR+CD123-CD11c-), and NK cells (CD3-CD20-CD8+CD16+). The percentage of activated cells (CD169+) within each subset was calculated. The gating strategy and definition of the different cellular subsets are shown. (TIF) [file pntd.0005637.s003.tif]

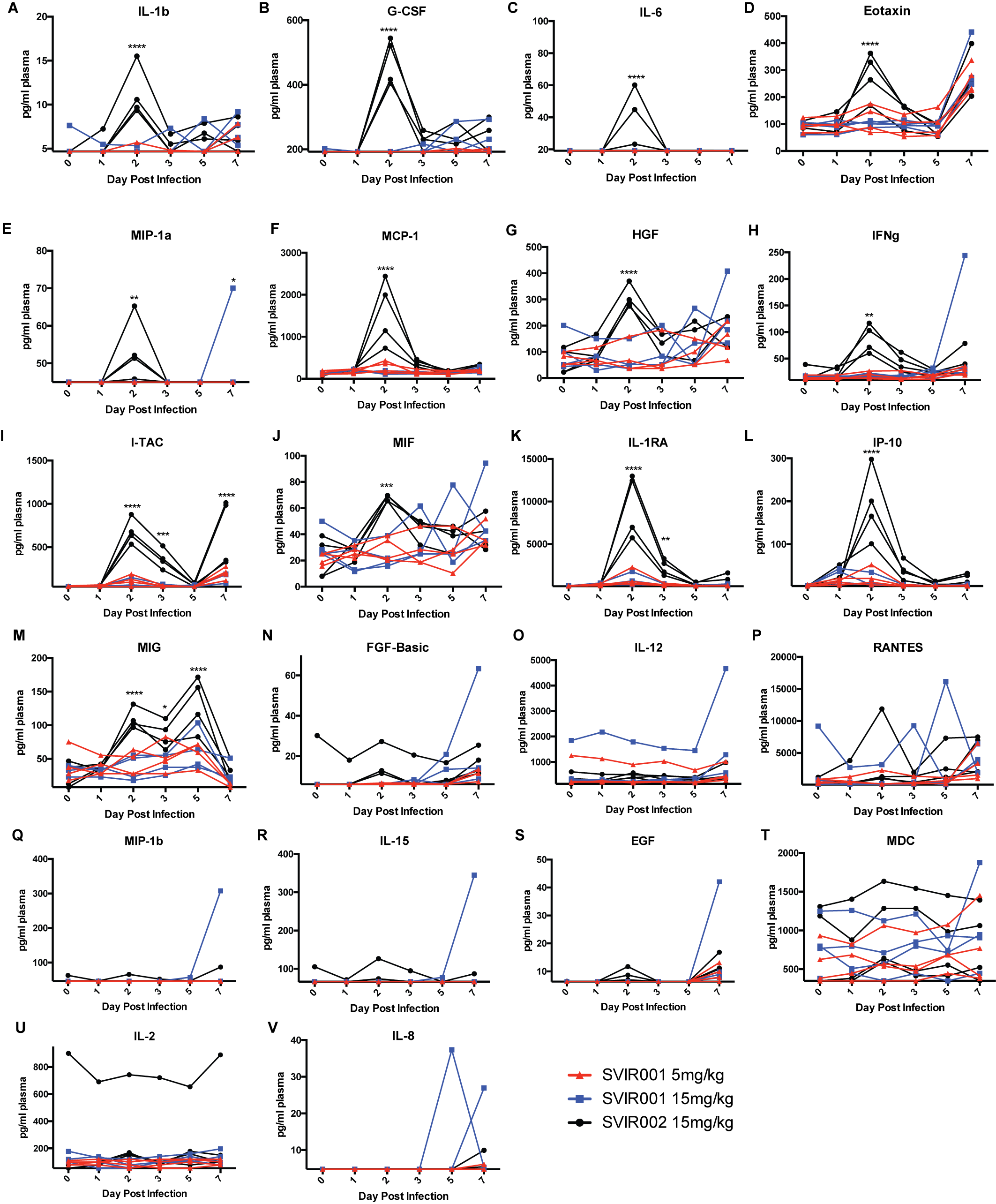

Supplement: S4 Fig — Cytokine analysis from 29-plex-cytokine magnetic bead assay was performed on plasma from animals treated with SVIR001 or control mAb SVIR002. Cytokine analysis revealed changes in plasma cytokine levels of (A) IL-1β, (B) G-CSF, (C) IL-6, (D) eotaxin, (E) MIP-1α, (F) MCP-1, (G) HGF, (H) IFNγ, (I) I-TAC, (J) MIF, (K) IL-1RA, (L) IP-10, and (M) MIG. Differences were analyzed using Sidak’s multiple comparison tests, and adjusted P values are reported (n = 4; ****, P < 0.0001, ***, P < 0.0005, **, P < 0.01, *, P < 0.05). Individual animals are graphed. Plasma cytokine levels of (N) FGF-Basic, (O) IL-12, (P) RANTES, (Q) MIP-1β, (R) IL-15, (S) EGF, (T) MDC, (U) IL-2, and (V) IL-8 did not demonstrate any significant changes between treatment groups. IL-10, IL-17, GM-CSF, VEGF, TNFα, and IL-4 remained below the limit of detection and are not shown. (TIF) [file pntd.0005637.s004.tif]

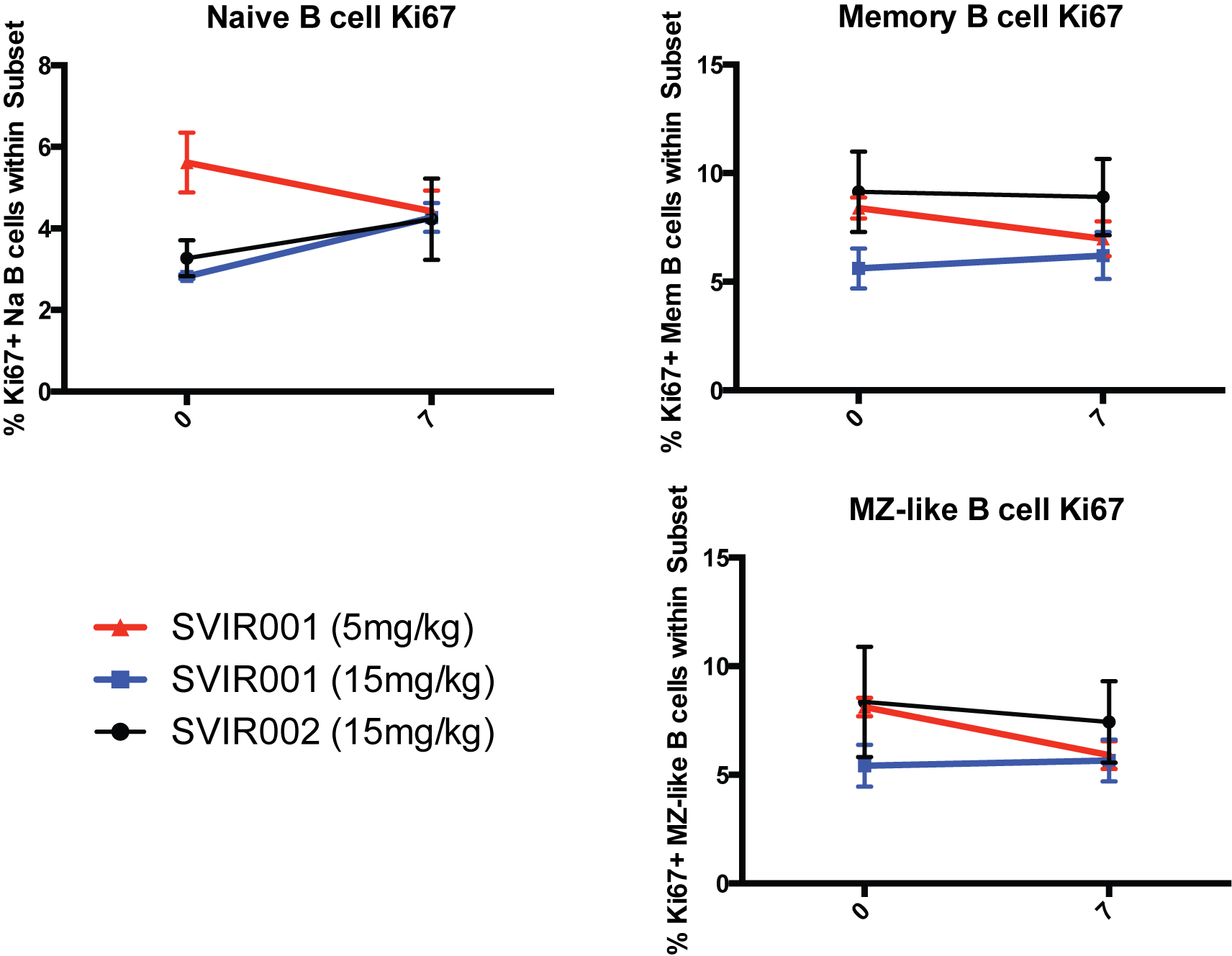

Supplement: S5 Fig — Total peripheral blood mononuclear cells were analyzed by flow cytometry for the presence of B cell proliferative responses following CHIKV infection in control and anti-CHIKV treated NHP. B cells were stained with antibodies directed against CD3, CD20, CD27, IgD and HLA-DR as well as Ki67 in order to identify proliferating (Ki67+) cells in naïve B cells, memory B cells and marginal zone like B cells. The percentage of actively proliferating cells within cell type was calculated using FlowJo software and the data was graphed in GraphPad Prism v6 software. (TIF) [file pntd.0005637.s005.tif]

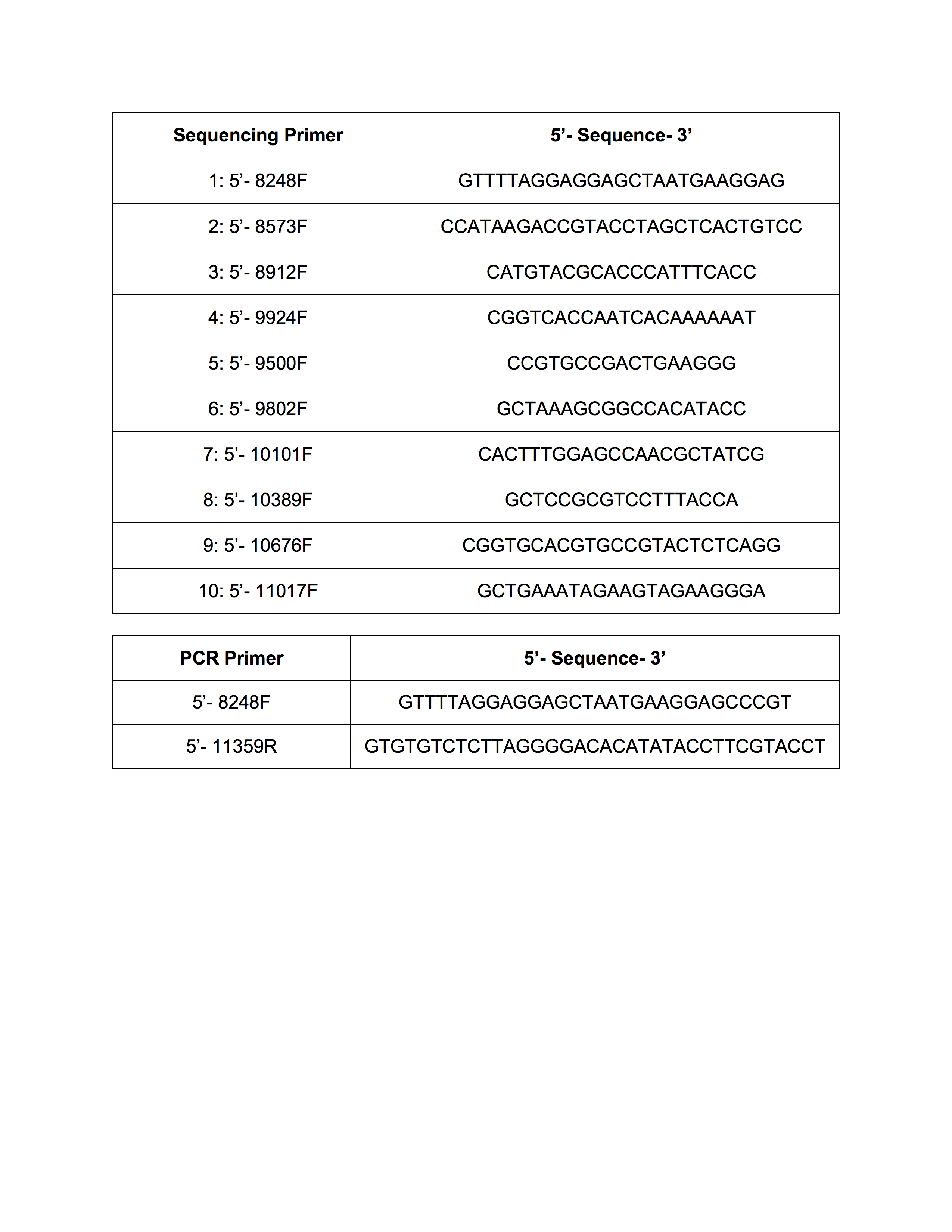

Supplement: S1 Table — (TIFF) [file pntd.0005637.s006.tiff]

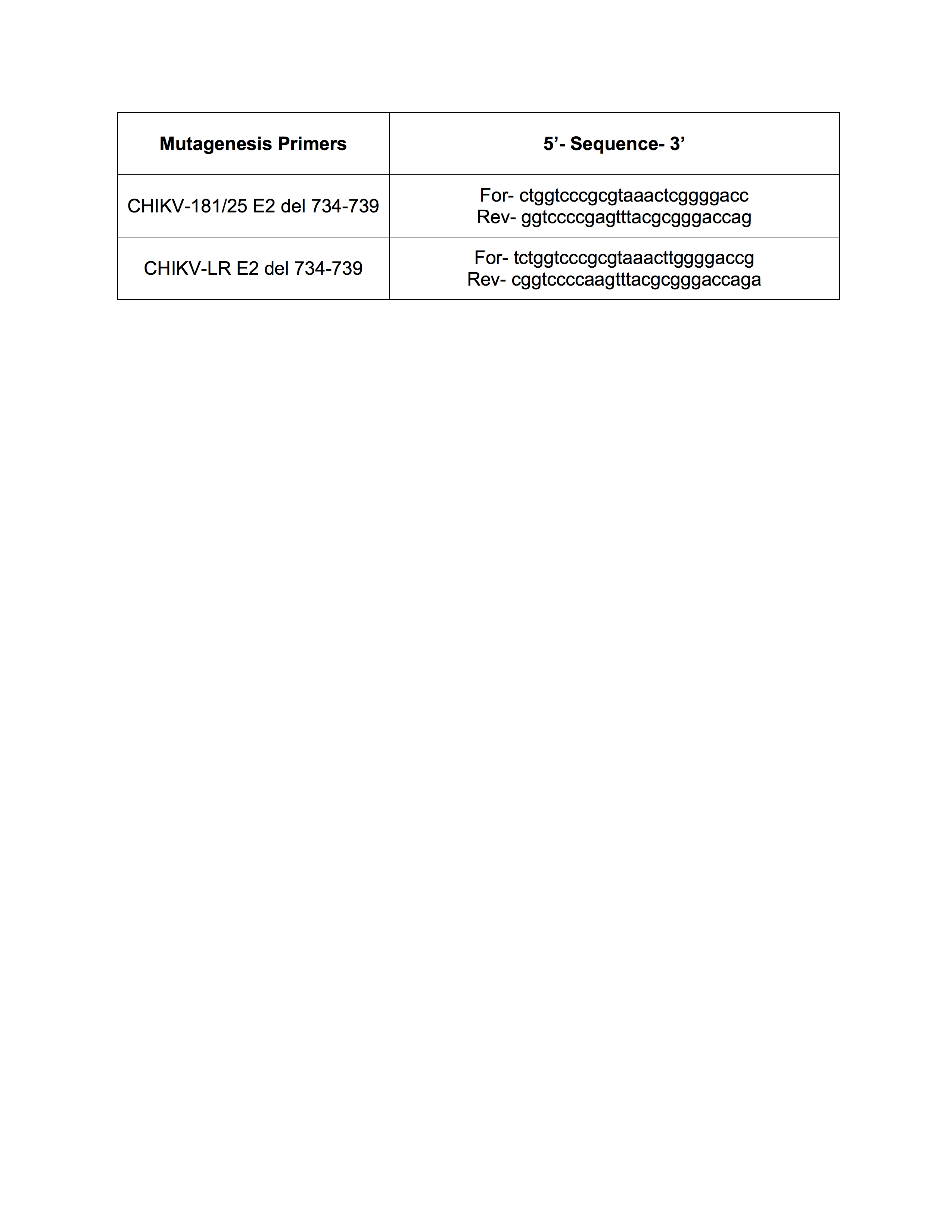

Supplement: S2 Table — (TIFF) [file pntd.0005637.s007.tiff]

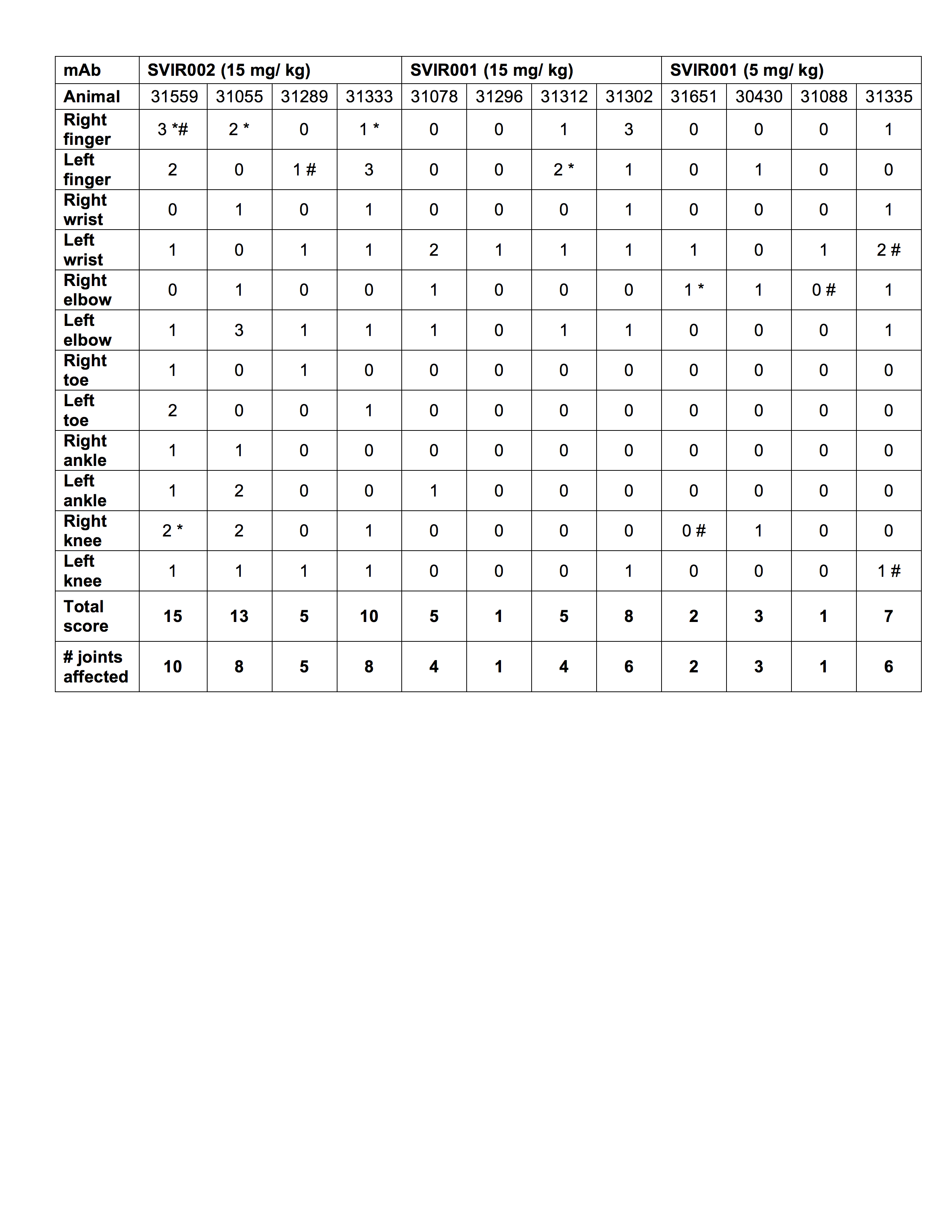

Supplement: S3 Table — H&E stained joint sections were scored as described in Table 2. Additional findings such as the presence of granulocytes or hemosiderin are indicated but were not used in the calculation of scores. * Granulocytes (eosinophils and/or neutrophils), # Hemosiderin (TIFF) [file pntd.0005637.s008.tiff]
